# Single-Molecule Detection Concepts Enabled by DNA Origami

**DOI:** 10.3390/mi17060741

**Published:** 2026-06-19

**Authors:** Seppe Driesen, Karen Leirs, Jeroen Lammertyn

**Affiliations:** Department of Biosystems, Biosensors Group, KU Leuven, Willem de Croylaan 42, 3001 Leuven, Belgium; seppe.driesen@kuleuven.be (S.D.); karen.leirs@kuleuven.be (K.L.)

**Keywords:** DNA nanotechnology, DNA origami, single-molecule, fluorescence, diagnostics

## Abstract

Since its introduction in 2006, DNA origami has enabled the fabrication of a wide variety of two- and three-dimensional DNA nanostructures. From the very beginning, researchers have explored these nanostructures as programmable nanobreadboards with hundreds of uniquely addressable positions, allowing precise spatial arrangement of biomolecules, fluorophores, and nanoparticles. This capability has been leveraged to create functional DNA nanomachines capable of single-molecule detection. Here, DNA origami is utilized to precisely engineer various nanoarchitectures, such as conformational switches and plasmonic hotspots. Through coupling of these concepts with tailored readout strategies, true single-molecule detection can be achieved. This literature review systematically examines the development of DNA origami-based single-molecule detection concepts. We first explore general design principles to produce functional DNA nanostructures, followed by an overview of non-fluorescence-based approaches employing atomic force microscopy, nanopores, and optical nanoantennas with surface-enhanced Raman spectroscopy readout, as well as fluorescence-based approaches relying on dynamic DNA nanostructures and optical nanoantennas with fluorescent readout. We highlight key trends as well as the remaining technology gaps that should be bridged to further advance DNA origami towards next-generation single-molecule detection.

## 1. Introduction

In bioanalytical measurements, the highest possible sensitivity and resolution is achieved by observing molecules and events at the single-molecule (SM) level [1]. Consequently, SM detection and analysis have become powerful tools across various research disciplines, including diagnostics and biophysics. In a diagnostic context, detection of individual target molecules, or very low amounts thereof, enables unmatched limits of detection (LODs) down to sub-femtomolar concentration levels. For many diseases, such sensitivity can facilitate earlier diagnosis, often leading to significantly improved treatment outcomes [2,3]. In the more fundamental setting of biophysics, there is also growing interest in platforms that enable not only the detection, but also the subsequent analysis of molecules with SM resolution. Such methods allow researchers to study complex systems in exceptional detail, uncovering hidden heterogeneities between individual molecules which are often masked in bulk analyses. Although these variations may be subtle, they often have significant biological implications and are therefore essential to fully understand biological processes, including protein–protein interactions, nucleic acid (NA)-protein interactions, and enzymatic activity [1,4].

In this context, DNA origami has emerged as a versatile platform to create nanostructures and -machines capable of SM detection and analysis. With DNA origami, virtually any two-dimensional (2D) or three-dimensional (3D) DNA nanostructure can be assembled with high yield [5]. The resulting nanostructures are often used as nanoscale molecular breadboards, where the staple sequences form an array of hundreds of uniquely addressable positions that can be functionalized with a precision of ~5 nm [6]. Using this principle, the simplest SM-sensitive nanosensor can be created by functionalizing a DNA nanostructure with a single bioreceptor. However, true SM sensitivity is only obtained by translating the resulting SM detection event into a measurable signal. DNA origami-based SM sensors therefore rely on smart nanostructure design combined with the appropriate readout methodologies.

**Figure 1:**
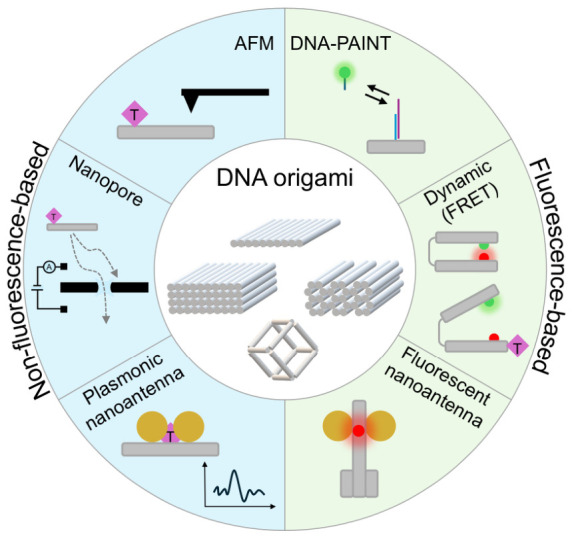
Overview of DNA origami-based SM detection methods discussed in this review. Non-fluorescence-based methods (blue) include the use of atomic force microscopy (AFM), solid-state nanopores, and optical nanoantennas with surface-enhanced Raman spectroscopy (SERS) as the readout mechanism. Fluorescence-based methods (green) include the combination of DNA origami with super-resolution microscopy like DNA points accumulation for imaging in nanoscale topography (DNA-PAINT), dynamic nanostructures detected with Förster resonance energy transfer (FRET), and optical nanoantennas with fluorescent readout.

Through the years, several reviews have excellently described DNA origami-enabled sensing from different perspectives [7,8,9,10,11]. In line with the review by Rajendran et al. from 2012 [7], this review specifically summarizes the advancements that have been made throughout the years in the development of DNA origami-based target detection concepts with SM sensitivity. First, we briefly introduce the general principles for turning DNA nanostructures into (SM) DNA nanosensors, followed by an overview of DNA origami-based SM detection concepts, categorized into non-fluorescence-based and fluorescence-based approaches, each with their own subcategories (Figure 1).

While DNA origami has also enabled a wide range of SM studies of intrinsic biophysical processes, such applications are not the primary focus of this review. Instead, we focus specifically on SM detection of external analytes, while studies in which DNA origami serves as a platform for biophysical measurements are only discussed when framed in a biosensing context. Over the years, these technologies have evolved from proof-of-concept methods to highly integrated concepts with a strong diagnostic focus. We discuss the strengths and limitations of the existing concepts and highlight opportunities for future research towards real-life applications.

## 2. Turning DNA Origami into SM Sensors

### 2.1. DNA Origami Design, Assembly, and Functionalization

**Figure 2:**
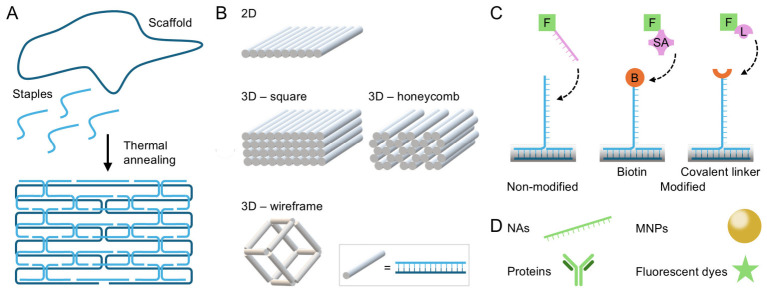
Overview of DNA origami design, assembly and functionalization. (**A**) In DNA origami, a long ssDNA scaffold sequence interacts with hundreds of short ssDNA staples in a thermal annealing procedure, resulting in a DNA nanostructure. (**B**) Virtually any 2D or 3D DNA nanostructure can be designed by connecting adjacent dsDNA helices (represented as grey cylinders) on a square or honeycomb lattice or a wireframe arrangement. (**C**) DNA nanostructures can be turned into functional nanomachines by implementing ssDNA staple extensions for attachment of functional moieties (indicated with F). These extensions can be non-modified for hybridization-based attachment or modified for other (non-)covalent attachment strategies such as biotin–streptavidin (indicated with B and SA) or covalent linkers (indicated with L). (**D**) Frequently used functional moieties include NAs, proteins, metallic nanoparticles (MNPs), and fluorescent dyes. These can be conjugated or modified to allow (non-)covalent attachment to the DNA nanostructure (as described in panel (**C**)).

The introduction of DNA origami by Paul Rothemund in 2006 represented a major breakthrough in the fabrication of DNA nanostructures. Before, DNA assemblies were typically limited to small, simple architectures, and assembly yields were generally low due to stoichiometric limitations. DNA origami enabled the construction of ~100 nm structures with high assembly efficiency by folding a long single-stranded (ss) DNA scaffold through hybridization with typically around 200 short ssDNA staples (Figure 2A) [5]. Since then, the design and fabrication protocols have been optimized and thoroughly documented, making the technology widely accessible across various fields of research [12,13,14]. Briefly, the process starts by designing the desired DNA nanostructure using specialized software. Here, the user decides between a 2D or a 3D geometry, and, in case of the latter, whether the design makes use of a square or a honeycomb lattice, or a wireframe arrangement (Figure 2B). Although caDNAno [12] has long dominated the field as one of the earliest and most widely adopted tools, a growing number of alternative platforms have emerged. Notable examples include scaDNAno [15], a user-friendly web-based caDNAno alternative; ENSnano [16] and MagicDNA [17] for complex 3D designs; and DAEDALUS [18] and PERDIX [19] for automated design of wireframe structures. These tools translate the design into DNA sequences by routing the scaffold DNA through the design, followed by generating the staple sequences that are required to assemble the structure. While the 7249-nucleotide (nt)-long M13mp18 viral genomic ssDNA has long served as the standard scaffold, recent advances enable the production of custom, design-specific scaffolds for increased versatility [20,21,22]. In parallel, alternative biological DNA sources, such as the ~50 kbp long λ-phage genome, have been explored for alternative DNA origami scaffolds to create larger DNA nanostructures [23,24]. The final origami design comprises an intricate arrangement of double-stranded (ds) DNA helices, where adjacent helices are connected by staple and scaffold crossovers. After the design process, the required sequences can be ordered from commercial suppliers. DNA nanostructures are then assembled via a thermal annealing procedure in a buffer environment with sufficient Mg^2+^ (typically 10–25 mM), followed by removal of excess staple sequences.

Beyond structural design, the mechanical properties of DNA origami nanostructures also influence their performance as SM sensors. Design choices (e.g., crossover density, helix packing, and overall architecture), as well as environmental conditions (e.g., ionic strength), govern the balance between structural rigidity and flexibility. Consequently, DNA origami designs can span a wide range from dense, multi-helix bundles to dynamic, reconfigurable systems incorporating hinges and strand displacement mechanisms [14,25]. Rigid designs are generally favored for applications requiring precise spatial control, such as those relying on Förster resonance energy transfer (FRET) and plasmonic coupling, as they minimize thermal fluctuations and positional uncertainty, thereby improving spatial accuracy and signal-to-noise ratios. In contrast, flexible and reconfigurable nanostructures enable conformational signal transduction and amplification, at the cost of increased structural heterogeneity and signal variability [26,27,28]. As a result, the design of DNA origami-based sensors involves a trade-off between structural stiffness and dynamic functionality, which should be optimized based on the intended readout mechanism and sensing application.

To finally transform these DNA nanostructures into nanomachines capable of SM detection, further functionalization and purification steps are performed to attach the desired functional moieties. Over the years, numerous methods have been developed for this purpose [29,30,31,32]. Typically, functionalization is achieved by incorporating ssDNA extensions at the 5′ or 3′ end of selected staple strands, causing them to protrude from the DNA origami surface. Depending on the type of functionalization to be implemented, the desired binding strength, and the application, these staple extensions can be (1) non-modified, for hybridization-based attachment, or (2) modified to allow non-covalent immobilization strategies such as biotin–streptavidin binding, or covalent strategies relying on click-chemistry or protein tags (Figure 2C) [31]. Early DNA origami studies already demonstrated application-oriented functionalization of DNA origami with, e.g., DNA (aptamers) [33], proteins [34], metallic nanoparticles (MNPs) [35], and fluorescent dyes [36] (Figure 2D). Also in the context of SM detection, these remain among the most frequently used functional moieties. From these, NAs (e.g., aptamers) are the most straightforward to incorporate, as they can be attached through hybridization or even by directly including the relevant sequence in the staple extension. For other functional moieties, conjugation to an NA for hybridization-based attachment or to linker molecules enabling (non-)covalent attachment is typically required.

### 2.2. SM Sensing and Assay Implementation

In this review, we adopt a broad definition of SM sensing in which individual target molecules bound to individual DNA nanostructures are directly observed or interrogated. Within this framework, a DNA origami nanostructure functionalized with a single bioreceptor can already be regarded as a basic SM sensor. However, practical SM sensing additionally requires conversion of single binding events into a measurable signal through an appropriate readout strategy. Because the choice of readout strongly influences sensor design, the studies discussed in this review are categorized based on the readout mechanism. Techniques such as atomic force microscopy (AFM) allow direct spatial visualization of individual nanostructures and bound molecules, particularly for larger targets. Similarly, SM surface-enhanced Raman scattering (SM-SERS) enables spectroscopic interrogation of individual nanostructures hosting single analyte molecules. In these cases, SM detection relies on probing individual nanostructure–target complexes, even when measurements are performed across populations of structures. For other readouts, such as fluorescence-based detection, additional labeling is typically required. Whereas single fluorophores can be detected with advanced optical setups, signal amplification may be required and can be directly integrated into the DNA nanostructure, facilitating SM detection. Importantly, although the approaches discussed here operate at the SM level according to the definition above, the extent to which individual binding events are resolved, temporally monitored, or digitally counted varies between studies.

Finally, from an application perspective, the SM nature of the detection approaches discussed in this review is not necessarily reflected in ultralow LOD values. Beyond SM detection capabilities, reported LOD values strongly depend on the overall assay format. For example, DNA origami-based SM sensors can be implemented in digital assays, where the binary signal from individual target binding events is evaluated across many individual reaction volumes, enabling up to attomolar detection limits [37]. However, although all studies discussed in this review are SM-sensitive according to the applied definition, many were intended as proof-of-concept demonstrations of SM sensing principles rather than fully integrated and ultrasensitive assays. Therefore, while LOD values are reported where available, they primarily serve as an indication of the application readiness of each approach and should not be used for direct comparison across all studies.

## 3. Non-Fluorescence-Based SM Detection Approaches

As a first category of DNA origami-enabled SM detection concepts, we introduce approaches that do not rely on fluorescence to observe the target binding event. Within this category, we discuss concepts based on AFM, nanopores, and optical nanoantenna SERS readouts (Table 1).

### 3.1. Atomic Force Microscopy

From the early days of DNA nanotechnology, AFM has been a widely applied characterization method, as it is one of the few methods enabling the direct visualization of DNA nanostructures [5,38,39]. AFM imaging is well-suited for high-resolution applications, as, depending on the experimental setup, spatial resolutions of ~1 nm or even lower can be reached [40]. When using AFM in the context of origami-based SM detection, two different approaches can be distinguished: (1) the direct observation of binding events onto DNA nanostructures, or (2) the observation of structural reconfigurations of dynamic nanostructures upon interaction with the target. While the former is routinely performed on simple, tile-like 2D DNA nanostructures, the latter often requires a slightly more intricate design.

**Table 1:** Overview of the non-fluorescence-based DNA origami-enabled SM detection concepts discussed in this review. For nanopore concepts, (P) and (A) respectively refer to concepts employing DNA origami attaching to the pore or the analyte. Concentration values refer to the applied concentration or the lowest concentration generating a response different from the blank. Underlined concentration values are LODs. Non-reported values are indicated with a horizontal dash. Concepts demonstrated in complex sample media were also demonstrated in buffer.

Ref.	Readout	Origami Design	Target Molecule	Lowest Reported Concentration or LOD	Demonstrated Sample Medium
[41,42]	AFM (direct)	2D	40 nt RNA	240 pM	Sample with high endogenous RNA content
			10–22 nt ssDNA	30 nM	Buffer
[33]	AFM (direct)	2D	Thrombin	12 nM	Buffer
[43]	AFM (direct)	2D	Streptavidin	20 nM	Buffer
[44]	AFM (direct)	2D	ssDNA	1 pM	Buffer
			RNA (after RT-PCR *)	-	Tap water, hospital environmental samples (soil, tap water, surface swabs), river water
[45]	AFM (reconfiguration)	3D	Streptavidin	8 nM	Buffer
			Anti-FAM IgG *	8 nM	
			Na^+^ (NaCl)	200 mM	
			K^+^ (KCl)	100 mM	
			Ag^+^ (AgNO_3_)	10 µM	
			miRNA-20, miRNA-16	200 nM	
			ATP	1 mM	
[46]	Nanopore (P)	3D	λ-DNA	1 nM	Buffer
[47]	Nanopore (P)	3D	Streptavidin	20 nM	Buffer
			IgG	20 nM	
			6 kbp * dsDNA	300 pM	
			50 nt ssDNA	300 pM	
			M13mp18 ssDNA	100 pM	
[48]	Nanopore (P)	3D	Holo-hSTf *	5 nM	Buffer
[49]	Nanopore (P)	3D	λ-DNA	1 nM	Buffer
			50 nt ssDNA	500 nM	
[50]	Nanopore (A)	2D	Human CRP *	3 nM	Buffer
				9 nM	5% human plasma
[51]	Nanopore (A)	2D	ATP *	1 mM	Buffer
[52]	Nanopore (A)	3D	miRNA-141-3p	2 nM	2% human serum
[53]	Nanopore (A)	2D	ssDNA, RNA	2 nM	Buffer
			miR-532, miR-21, let-7a, miR-221, miR-629	5 nM	Tissue and cell extracts
[54]	Nanoantenna (SERS)	2D	Streptavidin (alkyne-labeled)	20 nM	Buffer
[55,56,57]	Nanoantenna (SERS)	2D	Thrombin	20 nM	Buffer
			thioflavin T	100 pM	
			Epidermal growth factor receptor	200 pM	
[58]	Nanoantenna (SERS)	3D	Cyt c *	-	Buffer
			HRP *		
[59]	Nanoantenna (SERS)	3D	Streptavidin	300 nM	Buffer
			Thrombin	6.9 µM	

* Abbreviations in table that did not yet appear in the main text: RT-PCR = reverse transcription polymerase chain reaction, FAM = carboxyfluorescein, IgG = immunoglobulin G, bp = basepair, Holo-hSTf = holo human serum transferrin, CRP = C-reactive protein, ATP = adenosine triphosphate, Cyt c = cytochrome c, HRP = horseradish peroxidase.

#### 3.1.1. Direct Observation of SM Binding

**Figure 3:**
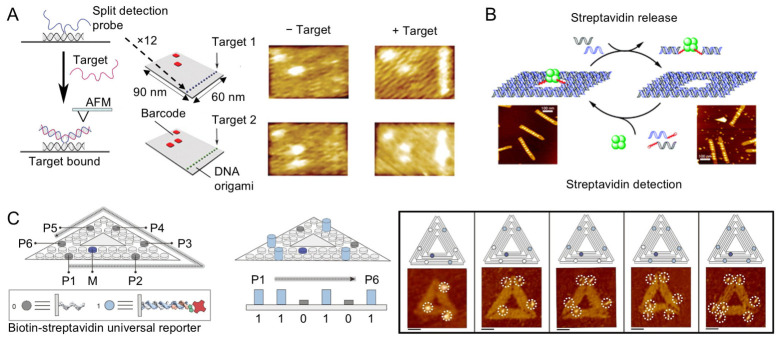
DNA origami-enabled SM detection with AFM for direct readout of target binding. (**A**) A split detection probe forms a rigid, V-shaped feature upon binding of target NA, which can be observed with AFM. Tiles with twelve of these probe pairs are barcoded to discriminate between different target sequences. Adapted with permission from Ref. [41], © 2008 The American Association for the Advancement of Science. (**B**) Streptavidin binding is directly observed on DNA origami with biotinylated binding pockets. Streptavidin can be released by adding the complement of the capture staple, followed by reintroduction of biotinylated staples to refresh the binding sites. Scale bars are 100 nm. Adapted with permission from Ref. [43], © 2010 The Royal Society of Chemistry. (**C**) Streptavidin binding to a biotinylated capture probe is used to detect NA target binding on a DNA nanostructure with AFM. Using six distinct capture probes at six positions (P1–P6), multiplex detection of (up to) six NA targets is demonstrated. A marker (M) is used to detect the orientation of the DNA origami. Scale bars are 40 nm Adapted with permission from Ref. [44], © 2026 the American Chemical Society.

In 2008, only two years after the introduction of the DNA origami technique, Ke et al. pioneered SM observation of NA target binding on DNA origami (Figure 3A) [41]. The authors designed DNA origami tiles with 12 pairs of ssDNA extensions on neighboring staple sequences, each pair forming a split detection probe. Upon interaction with the NA target, a rigid V-shaped structure was formed on the DNA origami surface, which could be directly visualized by AFM. Using barcoded tiles specifically developed for three murine gene sequences and a control sequence, multiplex RNA detection and RNA detection in a complex sample medium with high endogenous RNA content were successfully demonstrated. In a follow-up study, the DNA tiles were optimized to enable detection of ssDNA fragments as short as 10 nts, further expanding the versatility of DNA origami-based near-SM NA detection [42].

In a later work, the same group extended their approach to aptamer-based protein detection [33]. After successfully demonstrating the immobilization of up to eight proteins onto an individual DNA nanostructure [60], the authors used DNA origami to study the optimal interspacing between the 15-mer aptamer and the 29-mer aptamer for multivalent thrombin binding. Having proven that an approximate size match between the aptamer interspacing and the size of the protein maximized binding efficiency, six of these optimally interspaced aptamer pairs were placed on a DNA origami tile. As such, binding of up to six individual thrombin molecules per DNA origami could be observed with SM resolution [33], making this work one of the earliest examples of true SM protein detection on DNA origami nanostructures. Comparable results for SM protein binding were achieved by Numajiri et al., who demonstrated the selective binding of up to nine individual streptavidin molecules on a DNA origami with nine periodically interspaced streptavidin binding pockets functionalized with biotinylated staples (Figure 3B) [43]. Moreover, by including a toehold in these staples, the biotinylated capture strands could be selectively removed from the structure through a strand displacement process, releasing the captured streptavidin. Reintroduction of fresh capture strands restored the binding pocket to its original state, showcasing the full reversibility of the system.

More recently, in 2026, Xi et al. presented a novel, hybrid approach, developing a concept for multiplex detection of NA targets using streptavidin as a universal reporter for AFM readout [44]. A sandwich assay was proposed where NA targets related to six distinct bacterial species could bind to a capture probe at a specific position on a DNA origami nanostructure (Figure 3C). Biotinylated detection probes and streptavidin were then introduced to determine where the NA target had been bound. Using this system, the authors demonstrated (1) successful single- and multiplex detection of synthetic DNA target sequences at concentrations as low as 1 pM, (2) detection of up to 18 target NAs by producing trimer nanostructures, (3) reversibility of their system, using a strand displacement mechanism to remove previously bound target NAs and streptavidin, (4) detection of bacterial RNA, by implementing an intermediate reverse transcription polymerase chain reaction (RT-PCR) step, and (5) bacteria detection in complex environmental samples (i.e., samples collected in a hospital environment and river water).

While all of the concepts discussed here enabled observation of single molecules, many of these AFM-based approaches were primarily developed as proof-of-concept systems, demonstrating the feasibility of DNA origami as a platform for SM detection. Consequently, the reported target concentrations, typically in the high-picomolar-to-nanomolar range (Table 1), reflect experimental design choices rather than a strict focus on analytical sensitivity. Recent examples have demonstrated incorporation of features such as multiplexing and the use of universal reporter strategies [44], highlighting a shift towards more application-oriented designs. Nevertheless, several challenges remain for widespread applicability. For example, the detection of very small target molecules (e.g., adenosine triphosphate (ATP)) can be challenging to observe directly with AFM and thus requires different detection strategies. Also, this may be enabled through implementation of universal reporter strategies, as demonstrated by Xi et al.

#### 3.1.2. Observation of Structural Reconfiguration upon SM Binding

**Figure 4:**
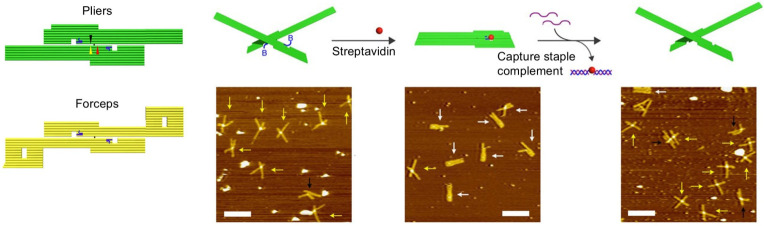
DNA origami-enabled SM detection with AFM for readout of structural reconfiguration. Pliers and forceps DNA nanostructures were developed that close upon binding with the target molecule (pinching), as shown here for streptavidin detection. The structures can be re-opened by addition of the complement of the capture staple. Scale bars are 300 nm. Adapted with permission from Ref. [45], originally published under CC BY-NC-ND 3.0 license.

By designing more complex, target-responsive DNA nanostructures, SM detection can be achieved through observation of structural reconfigurations of the DNA nanostructure upon target binding. Kuzuya et al. followed this principle to design SM beacons, responding to various chemical and biological target molecules (Figure 4) [45]. They developed a pliers and a forceps structure formed by two DNA origami arms that are connected at a central joint, consisting of a single DNA crossover. Both structures are functionally equivalent, but a different design of the arms enables visual discrimination in a multiplex detection setting. Target-induced closing of these DNA nanostructures (pinching) was successfully demonstrated by implementing specific ligands on one staple in each of the forceps or pliers arms: biotin for streptavidin detection, and carboxyfluorescein (FAM) for anti-FAM immunoglobulin G (IgG) detection. Notably, the pinching mechanism described here only works for multivalent target binding, as each target should interact with both arms to enable closing of the nanostructure. Strand displacement-mediated reversibility of streptavidin detection was also demonstrated, similar to the work discussed earlier for direct SM observation of streptavidin binding [43]. The specificity of the system was tested by incubating a mixture of biotin-labeled forceps and FAM-modified pliers with streptavidin, revealing nearly no closing of the pliers.

Using the same DNA origami, additional near-SM detection mechanisms were proposed to enable detection of monovalent targets or targets that exhibit weaker binding by implementing multiple target interaction sites. Metal ions were detected with a zipping mechanism, closing the structures upon cation-mediated DNA–DNA interactions. The presence of Na^+^ or K^+^ was detected based on G-quadruplex formation, where closing of the structure was shown through interaction with three or more cations. An alternative zipping mechanism was also introduced relying on Ag^+^-mediated stabilization of C-C mismatches in structures that carried interaction sites for eight Ag^+^-ions. With an unzipping mechanism, the authors demonstrated target-induced opening of the structure upon interaction with small molecules. In this strand displacement-based mechanism, the structure was closed with four dsDNA locks, containing an ssDNA overhang. This overhang was part of the detection probe: a complementary probe for micro RNA (miRNA) detection or an aptamer for ATP detection. With this mechanism, specific and multiplex detection of miRNA-20 and miRNA-16 was evaluated. Similarly, selective detection of ATP was demonstrated by incorporating four ATP aptamers, each binding two ATP molecules, and showed no cross-reactivity with guanosine triphosphate (GTP) [45]. A pH-responsive version of the DNA pliers was also developed [61].

Similarly to the examples with direct observation of target binding using AFM, these detection concepts were not developed with analytical performance as the primary objective, as reflected by the relatively high target concentrations reported (Table 1). Instead, they highlight the potential of AFM to directly visualize nanostructure–target interactions with SM resolution. From an application perspective, AFM-based approaches are less likely to be used as standalone sensing platforms, primarily due to the need for specialized instrumentation and low throughput. However, AFM remains an indispensable tool for characterization and validation, providing detailed insight into binding events, spatial organization, and structural reconfiguration. New studies using AFM as the primary readout method remain limited; also, the pliers structure discussed here was recently demonstrated with a luminescence-based readout [62], reflecting a broader transition toward more application-oriented approaches.

### 3.2. Nanopores

The second type of non-fluorescent strategy for DNA origami-based SM detection relies on nanopores. The passage of molecules through pores in membranes is an essential aspect of many biological processes. Inspired by nature, researchers have used this principle for the label-free investigation of charged polymeric molecules, such as NAs and proteins, translocating through nanopores, with SM resolution. This detection method relies on the resistive-pulse mechanism. When a voltage is applied over a membrane with nanopores connecting two reservoirs with an electrolyte solution, ion translocations generate a measurable ionic current. Passage of a molecule through the nanopore temporarily restricts ion flow by physically taking up part of the pore’s volume, resulting in a measurable current drop [63,64]. This signal forms a distinct fingerprint, where the duration, amplitude, and noise of the signal give information about the characteristics of the translocating molecule, such as its charge, size, shape, and affinity with the pore [65,66]. Additionally, these ionic current signals are also strongly influenced by the buffer composition and ionic strength (e.g., salt type, salt concentration, pH) [65].

While interesting results were achieved in the early days with biological nanopores such as α-haemolysin [67], these systems have several drawbacks, including limited stability and tunability, as well as small pore size. In this context, researchers have explored the fabrication of artificial, user-defined solid-state nanopores (SSNs) in various materials, including Si-based materials, glass, and graphene [63]. Moreover, various methods, including the use of DNA origami nanostructures, have been explored to tune SSN characteristics such as the pore size or the affinity for the target molecules [65,68,69,70,71]. In the following sections, we describe the two main strategies for the combination of DNA origami with SSN-based detection: (1) pore modifications, where DNA nanostructures with a central aperture can be used to control the pore size or to introduce functional groups at the pore opening, thereby modulating target affinity, and (2) DNA nanostructures passing through the SSN after interaction with analyte, resulting in a distinct molecular fingerprint [64].

#### 3.2.1. DNA Origami for Nanopore Modifications

**Figure 5:**
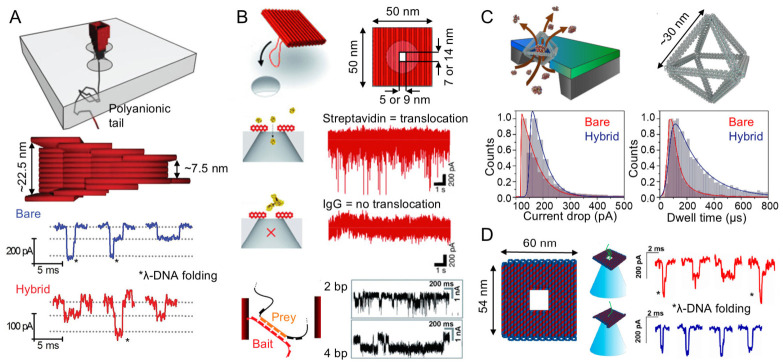
DNA origami-enabled SM detection with nanopores, with DNA origami being used for pore modifications. (**A**) A funnel-like DNA nanostructure is inserted into a SiN SSN, guided by its polyanionic DNA tail. This results in a reduced and more uniform pore diameter, showing less folding of λ-DNA upon translocation compared to the bare nanopore. λ-DNA folding is observed as a deeper, two-level current drop, indicated with *. Adapted with permission from Ref. [46], © 2011 the American Chemical Society. (**B**) A platelike DNA nanostructure is used as a lid (without an aperture) or as a size-selective gate on a SiN SSN. By tuning the pore size (9 nm × 14 nm), translocation of streptavidin is possible while IgG is blocked. By introduction of a DNA bait sequence, DNA preys can be stalled in the pore opening due to transient hybridization interactions. The increase in dwell time can be controlled by increasing the number of complementary bases between bait and prey. Adapted with permission from Ref. [47], © 2012 John Wiley and Sons. (**C**) DNA origami cages attached to the pore opening of a SiN SSN results in longer dwell times and deeper current blockades upon translocation of holo-hSTf due to non-specific interactions between the DNA origami and the target protein. Adapted with permission from Ref. [48], © 2024 the American Chemical Society. (**D**) Plate-like DNA nanostructures (similar to what is shown in panel (**B**)) attached to glass nanocapillaries behave the same way as they do on SiN SSNs. Adapted with permission from Ref. [49], © 2013 the American Chemical Society.

With DNA origami, user-defined nanopore modifications can be made, such as changing the size and shape of the pore opening and introducing analyte-specific functional groups or ligands in a controlled manner. In 2011, the group of Ulrich Keyser was among the first to explore this, developing hybrid nanopores by combining silicon-nitride (SiN) SSNs with 3D DNA origami (Figure 5A) [46]. In this proof-of-concept work, a funnel-like DNA nanostructure was created that reduced the nanopore’s opening and altered its shape from circular (with a diameter of 13–18 nm) to square (with a 7.5 nm edge). The geometry of the nanostructure enabled compatibility with a wide range of SSN diameters. To ensure its correct trapping in the SiN nanopore, a 2344-basepair (bp) nicked double helix was included as a polyanionic tail to guide insertion, inspired by earlier research using engineered α-haemolysin [72]. Upon translocation of λ-DNA, the authors observed current blockades consistent with typical λ-DNA translocation signatures. Moreover, because the DNA origami reduced the pore size, passage of folded DNA was less frequently observed for the hybrid nanopore compared to the bare nanopore, resulting in a more uniform signal [46].

In the lab of Hendrik Dietz, studies were performed using a simpler DNA origami that sits on top of the nanopore instead of being embedded inside. Their structure consisted of a 50 nm × 50 nm 3D DNA nanoplate of 6 nm thickness, packed on a honeycomb lattice (Figure 5B) [47]. Similarly to what was discussed before [46,72], nanoplate attachment to SiN nanopores with a diameter of 18–25 nm was guided by a 1300-nt ssDNA scaffold loop. The authors demonstrated that nanopore sensing could be controlled by implementing different-sized apertures at the center of the DNA nanoplate. When no aperture was included, the structure acted as a lid that attached stably onto the nanopore, producing a distinct current drop lasting for multiple hours. When an aperture was included, the structure acted as a size-selective molecular gate. Whereas streptavidin and IgG could freely pass through bare SiN nanopores, attachment of a nanoplate with a 9 nm × 14 nm aperture slowed down or even inhibited streptavidin and IgG translocation (Figure 5B). The authors also pioneered the use of DNA origami to incorporate target-specific bait sequences at the nanopore by adding staple extensions at the aperture. For translocation of 50-nt DNA preys, the bait sequences increase dwell time of the prey, resulting in a characteristic switching between two current levels. Increasing the complementarity from two to four bases further increased the dwell time. This concept was extended to longer and more complex DNA targets (M13mp18 scaffold DNA) and by including more bait sequences, yielding similar results with more complex current fingerprints [47]. Recently, Joty et al. reported a hybrid nanopore with a completely different geometry. In this work, a 3D octahedral DNA origami cage was constructed and inserted into a SiN SSN with one of its vertices (Figure 5C). Upon translocation of holo human serum transferrin (holo-hSTf) through the hybrid nanopore, deeper current blockades and longer dwell times were observed, due to non-specific interactions between the protein and the DNA origami cage. As a result, the hybrid nanopore showed an increased sensitivity for holo-hSTf compared to the bare nanopore [48].

Other studies extended the technology of DNA origami hybrid nanopores to alternative SSN materials. In 2013, the Keyser lab reported the construction of hybrid nanopores combining DNA origami with glass nanocapillaries, which are significantly easier and cheaper to produce than SiN nanopores (Figure 5D) [49,73]. DNA origami is particularly useful here because the fabrication process of glass nanocapillaries typically results in slightly larger and more variable pore diameters compared to SiN nanopores. Attachment of a DNA origami structure at the pore opening yields glass–origami hybrid nanopores with more uniform pore sizes. Experiments with these hybrid nanopores confirmed earlier results from SiN–origami hybrid nanopores: (1) plate-like DNA origami nanostructures can attach stably and reversibly to the glass nanocapillary, (2) λ-DNA exhibits reduced folding when translocating through smaller, hybrid pores, and (3) DNA bait sequences can stall ssDNA preys in a length- and sequence-dependent manner [49].

Notably, similarly to the AFM-based concepts, the studies discussed here also focused on demonstrating the fabrication and operation of functional hybrid nanopores with DNA origami, rather than detection of extremely low target concentrations (Table 1). Nevertheless, nanopore readouts are intrinsically SM, and SSN-based detection down to femtomolar and even attomolar concentrations has been reported [74,75,76]. As such, it is not unlikely that similar sensitivity may eventually be achieved with origami-based hybrid nanopores. Moreover, SSNs are widely studied, and translation to DNA origami-based hybrid nanopores towards real-life applications may therefore benefit from the availability of a well-established technological framework.

#### 3.2.2. DNA Origami Interacting with the Analyte Prior to Translocation

In an alternative nanopore-based approach, DNA origami is not used to alter the nanopore aperture, but as a structure carrying or interacting with the target analyte passing through the pore. Such approaches are especially interesting for nanopore-based analysis of small target molecules that typically pass too quickly through the nanopore to enable accurate detection [52,77]. Additionally, by designing DNA carrier molecules for multiple target analytes, each resulting in a distinct ionic current fingerprint, different analytes are easily distinguished from each other and from sample matrix molecules. Importantly, all of this is possible while keeping the nanopore as simple as possible, i.e., a single SSN can specifically detect multiple target analytes without requiring any modification or functionalization of the pore [69]. Following this principle, concepts have been developed for the SM detection of dsDNA structural motifs [78,79,80,81,82], proteins [79,83,84,85,86], and even viral RNA targets in clinical samples [87] using linear DNA carrier molecules. While these studies with linear carriers were not using DNA origami nanostructures, they relied on key DNA origami principles by using a linearized M13 scaffold hybridized with short, complementary oligonucleotides to form a nicked, double-helical construct. As such, they helped establish the conceptual groundwork for later studies using DNA origami. Just like linear dsDNA constructs, the trapping and translocation of DNA nanostructures also result in distinct current fingerprints [82,88,89,90,91,92,93,94]. DNA nanostructures may even result in more robust signal generation, as they are generally more rigid, thus reducing the frequency of false positives that may be observed due to folds, loops, and knots that may appear in linear dsDNA constructs [52].

**Figure 6:**
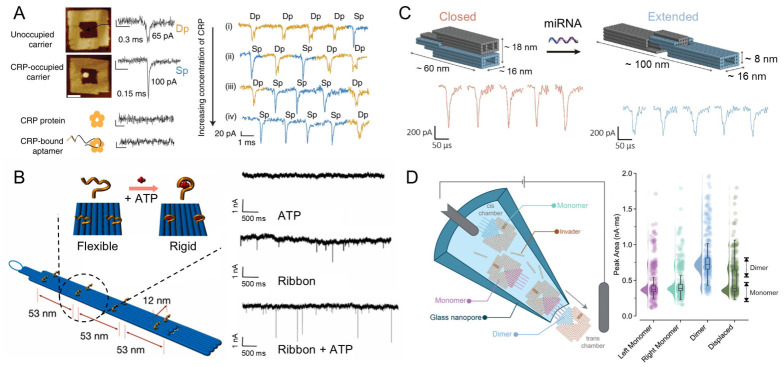
DNA origami-enabled SM detection with nanopores, with DNA origami being used for analyte modifications. (**A**) DNA origami nanoframes with or without aptamer-bound CRP show a single peak (Sp) or double peak (Dp), respectively, upon nanopore translocation, while aptamer-bound and free CRP on their own cannot be detected. Single peaks are increasingly observed for increasing CRP concentrations. Scale bar is 30 nm. Adapted from Ref. [50], originally published under CC BY 4.0 license. (**B**) DNA origami ribbons functionalized with ATP aptamers enable specific ATP detection. Translocation of ATP shows no signal, while small and large peaks are observed upon translocation of the ribbon without or with ATP, respectively. Little to no signal is observed for the ribbon upon addition of GTP or CTP. Adapted with permission from Ref. [51], © 2022 Elsevier. (**C**) The closed and extended configuration of a hinge DNA nanostructure each result in a distinct current fingerprint upon translocation through an SSN. The hinge can be opened through specific binding of miRNA-141-3p. Adapted from Ref. [52], originally published under CC BY 4.0 license. (**D**) DNA origami monomers (~80 nm side length) and dimers show a distinct signal upon SSN translocation. The target sequence acts as an invader that can break the link between the dimers through a strand displacement mechanism. Adapted from Ref. [53], originally published under CC BY 4.0 license.

The use of DNA origami as carriers was reported for the first time by the lab of Paolo Actis in 2020, who used aptamer-functionalized 2D DNA origami frames for the detection of human C-reactive protein (CRP) with glass nanopores (Figure 6A) [50]. Based on earlier observations that the translocation of similarly sized DNA origami frames and tiles through a nanopore results in distinct current fingerprints, a double peak or a single peak [90], respectively, the authors hypothesized that a similar effect could be obtained with proteins occupying the central cavity of a DNA frame. By employing a large nanopore (100 nm diameter), the researchers eliminated background signal from sample matrix molecules or unbound CRP, while successfully distinguishing unoccupied from CRP-occupied DNA nanoframes. The system exhibited good sensitivity (LOD 3 nM) and specificity (i.e., no CRP binding to non-specific aptamer, and no non-specific binding to CRP aptamer), and enabled CRP detection in 5% human plasma, with a slightly higher LOD (9 nM) [50]. In 2022, Ding et al. reported similar results using a ribbon-like DNA origami geometry [51]. Translocation of an aptamer-functionalized 2D DNA origami through a SiN nanopore resulted in distinct ionic current signatures when ATP was bound, while ATP on its own could not be detected (Figure 6B). Additionally, it was shown that the observed current signature depended on the aptamer arrangement on the nanoribbon. The researchers also used SSN readouts to demonstrate excellent specificity of their system (limited background signal in presence of GTP or cytidine triphosphate (CTP)) and to observe enzymatic conversion of aptamer-bound adenosine monophosphate into inosine monophosphate by adenosine deaminase.

The combination of nanopores with DNA origami was also demonstrated for NA targets (ssDNA, RNA, miRNA). 3D DNA origami hinges that undergo structural reconfiguration upon target detection were combined with quartz nanopores for the detection of miRNA-141-3p, a prostate cancer biomarker, in 2025 (Figure 6C) [52]. The hinge structure was designed such that the extended state was preferred, but the closed state was maintained in the absence of a target. Upon interaction with the target miRNA (and helper sequences), a mousetrap-like mechanism was triggered, converting the hinge from its closed to its extended state. Both states resulted in distinct current fingerprints when translocating through the nanopore, enabling sensitive and specific miRNA detection down to nanomolar concentrations in buffer as well as in 2% human serum. Finally, the Actis group recently reported a different strategy for NA detection. Here, interaction with target sequences induces a strand displacement mechanism that splits a DNA origami dimer into two monomers that can be distinguished based on their translocation fingerprints (Figure 6D) [53]. This concept was successfully demonstrated for ssDNA and RNA, including multiplex miRNA detection. Importantly, as the strand displacement interaction is nearly irreversible, the concept is highly robust and insensitive to RNase contamination, with the authors even showing the use of RNase digestion as a sample cleanup method prior to SSN translocation.

Compared with previous studies in which DNA origami nanostructures were attached to the SSNs (see Section 3.2.1), the work discussed here places greater emphasis on assay-oriented performance and operation in complex sample matrices (Table 1). This reflects a difference in research focus. Studies on nanopore modification are technology-driven, aiming to tune pore properties by creating DNA origami-SSN hybrid nanopores. In contrast, analyte-focused studies often start from one specific molecular target and aim to use DNA origami for tailored capture efficiency and target-specific readout. From an application perspective, such analyte-focused designs are more readily integrated into practical nanopore workflows, while still benefiting from established technological concepts.

### 3.3. Plasmonic Nanoantennas

The third and final type of non-fluorescent strategy for DNA origami-based SM detection relies on optical nanoantennas. Optical nanoantennas are structures capable of amplifying optical signals, which can be fabricated by placing two MNPs in close proximity, creating a plasmonic hotspot in the interparticle gap. Apart from the influence of environmental conditions, such as buffer composition and ionic strength [95], their performance depends on several parameters, including the particle material, shape, and size, as well as the interparticle distance. Additionally, the amplification efficiency also depends on the precise placement of the molecule of interest close to the MNPs [96]. Therefore, to ensure adequate and reproducible amplification, such nanoantennas should be fabricated with nanoscale precision [97,98,99]. While controlled fabrication can be achieved with top-down nanofabrication techniques, these methods are often expensive, complex, and labor-intensive [100]. In this context, DNA origami nanostructures functionalized with MNPs are increasingly explored as nanoantennas, taking optimal advantage of the nanoscale programmability of DNA origami to control the placement of MNPs and bioreceptors [101]. These DNA-origami nanoantennas have been used for SM detection concepts with various readout methods, including fluorescence (see Section 4.2) and SERS [101,102]. Raman scattering is a fundamental light–matter interaction in which photons are inelastically scattered, i.e., the scattered photons have a different energy and direction than the incident photons. The scattered light can be analyzed as a Raman spectrum, providing a characteristic vibrational fingerprint of the molecule under study. Because Raman signals are typically weak, SERS exploits localized surface plasmon resonances (LSPRs) in nanostructured noble metals, such as gold and silver, to achieve orders-of-magnitude signal enhancement [103,104]. SM-SERS enables the detection of Raman spectra from individual molecules by leveraging plasmonic hotspots, which, as discussed above, can be achieved with DNA origami nanostructures in combination with MNPs [101].

**Figure 7:**
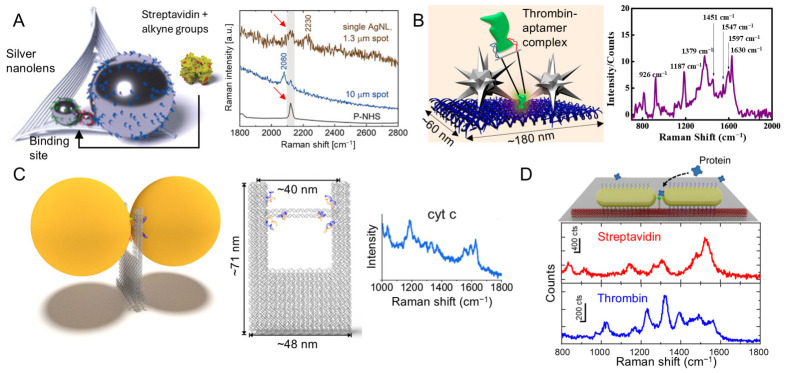
Optical nanoantennas with SERS readout for SM detection, fabricated using DNA origami. (**A**) A silver nanolens is created by attaching AgNPs of 10, 20, and 60 nm to a triangular DNA origami (~120 nm sides). Immobilization of alkyne-modified streptavidin in the hotspot between the smallest AgNPs reveals a distinct alkyne peak (red arrow) in the recorded Raman spectra (brown = nanolens, black = reference alkyne molecule). Adapted with permission from Ref. [54], © 2018 John Wiley and Sons. (**B**) Bimetallic Ag-coated Au nanostars are immobilized on a rectangular DNA origami dimer, enabling aptamer-based SM detection of thrombin. Adapted with permission from Ref. [55], © 2021 the American Chemical Society. (**C**) A DNA origami nanofork structure was introduced as a more flexible concept, for detection of cytochrome c (cyt c) and horseradish peroxidase. Adapted with permission from Ref. [58], originally published under CC BY-NC-ND 4.0 license. (**D**) Nanoantennas employing Au nanorods on DNA origami (~215 nm long) are proposed as a solution to create larger hotspots, allowing protein capture after nanoantenna assembly, while still ensuring sufficient signal amplification. This was demonstrated for streptavidin and thrombin detection. Adapted from Ref. [59], originally published under CC BY 4.0 license.

In 2013, the group of Ilko Bald pioneered the use of DNA origami to precisely position two gold nanoparticles (AuNPs) in close proximity to create a plasmonic hotspot for SM-SERS [105]. Since then, many others have reported on the fundamental aspects of DNA origami-enabled SM-SERS [106,107,108,109,110,111,112,113,114,115,116,117], as recently reviewed by Niu et al. [101]. SM detection using such concepts was first reported in 2018, also by the group of Ilko Bald, for the selective capturing of individual streptavidin molecules within a plasmonic hotspot (Figure 7A) [54]. Here, silver nanoparticles (AgNPs) of 10, 20, and 60 nm were immobilized on a DNA origami nanostructure to create a silver nanolens, resulting in a cascaded amplification with the strongest field between the smallest MNPs. Immobilization of streptavidin within this gap resulted in a distinct SERS spectrum upon excitation at 532 nm. While this work represents the first step to SM-SERS sensing enabled by DNA origami, direct sensing applications were not yet possible with this approach, as streptavidin had to be chemically modified with alkynes to overcome the background signal of DNA. A Raman peak, originating from the triple bonded C-C in these alkyne groups, was then used to detect successful immobilization of streptavidin. Moreover, the AgNPs were only attached after streptavidin immobilization, further limiting practical applicability. Later, the group of Tapasi Sen demonstrated a DNA nanoantenna consisting of Ag-coated Au nanostars immobilized on 2D DNA origami dimers for sensitive, label-free detection of pyocyanin and dopamine, and SM detection of thrombin (Figure 7B) [55,118,119]. These studies greatly enhance the application potential of the concept by omitting chemical modification of the analyte. Moreover, by using bimetallic MNPs, the nanoantenna exploits the optimal plasmonic properties of Ag together with the more desirable chemical properties of Au [54,120]. For SM thrombin detection, two thrombin-binding aptamers were placed within the 10 nm interparticle gap, matching the optimal inter-aptamer distance described by Rinker et al. [33]. Upon 532 nm laser excitation, the authors were able to observe individual thrombin molecules immobilized on the nanoantennas, resulting in a characteristic SM-SERS spectrum for thrombin. No non-specific signal was observed upon incubation with bovine serum albumin (BSA) or myoglobin [55]. The authors later explored the same DNA origami design with different MNP geometries. With an origami-based nanoantenna where the plasmonic hotspot was created between two Au nanobipyramids, sensitive and specific aptamer-based detection of the neurodegenerative disease biomarker thioflavin T was demonstrated down to an LOD of 0.1 nM [56]. Similarly, with a plasmonic hotspot between Au nanorods on an aptamer-functionalized origami dimer, a cancer biomarker, epidermal growth factor receptor, was detected down to a concentration of 0.2 nM [57].

In 2021, the Bald group also explored label-free SM-SERS detection of proteins using a 3D DNA origami nanofork antenna structure (Figure 7C) [58]. This nanoantenna was used for SM detection of cytochrome c (cyt c) and horseradish peroxidase (HRP) proteins. Non-covalently bound cyt c and covalently bound HRP within the plasmonic hotspot both resulted in Raman spectra showing characteristic peaks for the presence of heme groups upon excitation at 633 nm. This nanoantenna design holds great promise due to its inherent flexibility, demonstrated by the implementation of both AuNPs and AgNPs, a tunable interparticle gap size, and SM-SERS detection of proteins as well as small molecules [58]. The same nanofork structure was later also used for SM analysis of HRP enzyme activity [121], and hemin [122].

One issue often observed in SM-SERS nanoantennas is that the size of typical proteins exceeds the optimal interparticle distance for efficient amplification (1–2 nm), complicating protein capture in the hotspot [54,58]. As a result, the hotspots of most nanoantenna geometries are not readily accessible. Even for the nanofork which has a tunable interparticle gap, the issue remains, and the nanoantenna requires capturing of the analyte before assembling the MNPs onto the structure [58], limiting practical applicability. Solving this problem by ensuring that the plasmonic hotspot is sufficiently large and accessible while still capable of strong SERS amplification would therefore be highly valuable, as proposed by Schuknecht et al. (Figure 7D), and which was achieved by using Au nanorods instead of AuNPs, because they have an increased tip-curvature-to-volume ratio and decreased plasmon damping [59]. Implementing a single biotin or a thrombin-binding aptamer enabled detection of streptavidin and thrombin, respectively, resulting in Raman spectra showing indicative peaks of the proteins under study.

While these SM-SERS sensing concepts enabled by DNA origami have clearly shown their potential, there are several challenges to overcome towards real practical applicability [123]. While SERS spectra present a detailed molecular fingerprint of the analyte, this also has an inherent drawback, as these complex signals require thorough interpretation, unlike simple yes/no signals. The readout is even further complicated by background signals arising from the DNA nanostructure, the capturing strands or molecules, and sample matrix molecules. Nevertheless, SM-SERS remains a powerful technique that, from an application perspective, may be better positioned as an SM analytical platform. Practical implementation as a method for SM analyte detection would require thorough simplification of experimental and analysis workflows. However, the development of DNA nanoantennas, including those with other readout modalities, remains a prominent area of focus within DNA origami-enabled SM detection.

## 4. Fluorescence-Based SM Detection Approaches

The second category of origami-based SM sensing relies on fluorescence. Although fluorescence is not the only viable readout, as demonstrated above, it is often the preferred technique for SM detection concepts, thanks to its high sensitivity, specificity, and compatibility with biological environments [124,125]. As such, SM fluorescence microscopy has become a powerful tool over the last 30 years for both biophysics research, and, more recently, biomolecular detection. In the following sections, we discuss three major classes of fluorescence-based SM detection concepts enabled by DNA origami: those employing super-resolution microscopy techniques, dynamic DNA nanostructures, and DNA nanoantennas (Table 2).

### 4.1. Super-Resolution Microscopy

**Figure 8:**
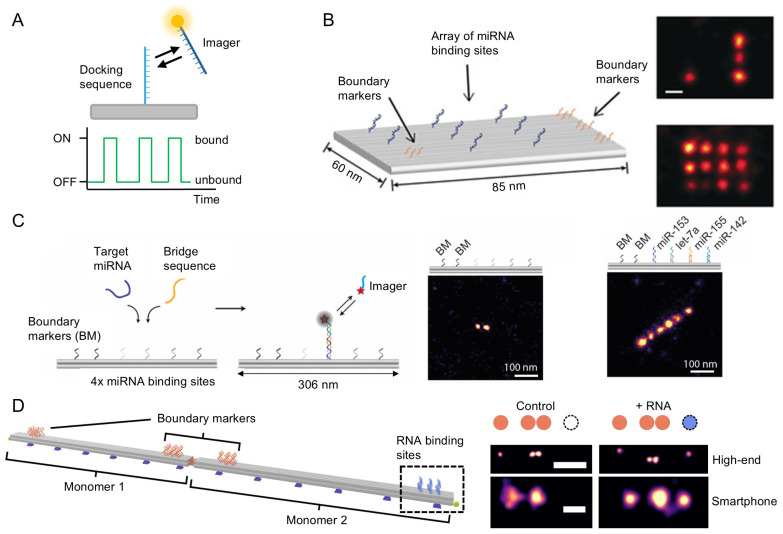
SM sensing with DNA origami using DNA-PAINT. (**A**) In DNA-PAINT, super-resolution originates from the transient binding and unbinding of an imager sequence at a docking sequence, resulting in a blinking signal. (**B**) An array of miRNA binding sites was created on a 2D rectangular DNA origami. The rotational position of the structure was resolved using 4 boundary markers at the edges. Using DNA-PAINT, up to 8 cancer-related miRNAs were detected. Adapted with permission from Ref. [133], © 2018 John Wiley and Sons. (**C**) A row of miRNA binding sites was created on an 8 HB DNA origami. By using a bridge sequence, all miRNAs could be detected with the same imager sequence. Using DNA-PAINT, up to 4 cancer-related miRNAs were detected. Adapted with permission from Ref. [134], originally published under CC BY-NC-ND 4.0 license. (**D**) Using a dimer of the 8 HB DNA nanostructures, the distance between the boundary markers and the binding sites was increased. Ebola RNA was successfully detected on a high-end microscope and on a smartphone-based microscope with lower resolution. Adapted from Ref. [135], originally published under CC BY 4.0 license.

Soon after its introduction, DNA origami was combined with super-resolution microscopy, leveraging its nanoscale addressability. Early work proposed DNA origami as a calibration standard for SM high-resolution imaging with photobleaching (SHRImP), direct stochastic optical reconstruction microscopy (dSTORM), and blink microscopy [36]. Later studies shifted almost entirely to DNA points accumulation for imaging in nanoscale topography (DNA-PAINT) as the preferred super-resolution technique [126,127,128,129]. In DNA-PAINT, super-resolution is achieved by imaging the transient binding of weakly interacting (i.e., short) imager strands to their complementary docking strands (Figure 8A). The resulting blinking can then be used to reconstruct a high-resolution image of the object under study. This method has found widespread use as a super-resolution technique, with dedicated software (Picasso) and comprehensive protocols for various implementations, such as its combination with antibody labeling, quantitative PAINT (qPAINT), and Exchange-PAINT for multiplexing, and resolution enhancement by sequential imaging (RESI), enabling Ångström-scale resolution [128,130,131,132]. Whereas many studies have shown the combination of DNA origami and DNA-PAINT as a benchmarking technique, later work also established this combination for SM sensing applications.

**Table 2:** Overview of the fluorescence-based DNA origami-enabled SM detection concepts discussed in this review. Concentration values refer to the applied concentration or the lowest concentration generating a response different from the blank. Underlined concentration values are LODs. Non-reported values are indicated with a horizontal dash. Concepts demonstrated in complex sample media were also demonstrated in buffer.

Ref.	Readout	Origami Design	Target Molecule	Lowest Reported Concentration or LOD	Demonstrated Sample Medium
[133]	Fluorescence (PAINT)	2D	16 miRNA targets	100 fM	Buffer
			Multiplex 8 miRNA targets	-	HeLa RNA extract
[134]	Fluorescence (PAINT)	3D	miR-153, let-7a, miR-155, and miR142 (+multiplex)	11 fM	Buffer
			Multiplex miR-21 + let-7a	-	MCF-7, HeLa, and MDA-231 RNA extracts
			miR-21, miR-142, let-7a	-	Patient plasma samples
[135]	Fluorescence (PAINT)	3D	30 nt RNA (Ebola)	~330 nM	Buffer
[136]	Fluorescence (FRET)	3D	23 nt ssDNA	100 pM	Buffer
[137]	Fluorescence (FRET and FQ *)	3D	miRNA-153 DNA analog (ODN-153)	1.6 pM (3.3 pM with FQ)	Buffer
			miRNA-342 DNA analog (ODN-342)	1 pM (3.9 pM with FQ)	
			Multiplex ODN-153 + ODN-342	-	
			Mutliplex miRNA-21 + let-7A	-	miRNA extracted from MCF-7 breast cancer cells
[138]	Fluorescence (FRET)	3D	17 nt ssDNA	1 nM	Buffer
			Anti-Dig * antibody	10 pM	50% human plasma
			PDGF-BB *	100 pM	Buffer
			Xhol restriction enzyme	66 units/mL	Buffer
			36 nt crRNA *	22 nM (assembled Cas9 RNP * complex)	Buffer
			Multiplex anti-Dig + anti-DNP *	-	Buffer
			Multiplex Xhol + 17 nt ssDNA	-	Buffer
[139]	Fluorescence (FRET and FQ)	3D	26 nt ssDNA	0.71 nM (bulk)	Buffer
[140]	Nanoantenna (fluorescence)	3D	9 nt ssDNA (ATTO655-labeled)	100 nM	Buffer
[141]	Nanoantenna (fluorescence)	3D	23 nt ssDNA (Zika)	1 nM	Heat-inactivated human serum
			23 nt RNA (Zika)		Buffer
			Multiplex 23 nt ssDNA (Zika) + 30 nt ssDNA		Buffer
[142]	Nanoantenna (fluorescence)	3D	34 nt ssDNA (*K. pneumoniae*)	2 nM	Heat-inactivated human serum
[143]	Nanoantenna (fluorescence)	3D	Anti-Dig antibody	1 nM	Buffer
[144]	Nanoantenna (fluorescence)	3D	151 nt ssDNA (*K. pneumoniae*)	~5 aM	Buffer
				~10 aM	Human plasma

* Abbreviations in table that have not yet appeared in the main text: FQ = fluorophore-quencher, Dig = digoxigenin, PDGF-BB = platelet-derived growth factor BB, crRNA = CRISPR-RNA, Cas9 RNP = CRISPR-associated protein 9 ribonucleoprotein, DNP = dinitrophenyl.

DNA-PAINT-based SM sensing on DNA origami was first demonstrated in 2018 by Xu et al. for multiplex miRNA detection [133]. A DNA nanostructure was developed carrying an array of ssDNA capture probes with 20 nm interspacing (Figure 8B). These probes were designed to stably capture their miRNA targets, while leaving a part of the target as a short, ss overhang for DNA-PAINT imaging. By implementing boundary markers on the DNA origami, its orientation could be determined accurately, enabling multiplex miRNA detection. With this concept, singleplex detection of 16 and multiplex detection of 8 cancer-related miRNA targets was demonstrated, as well as detection of miRNA in HeLa cell extracts. Absence of signal upon incubation with single-base mismatched targets indicated excellent specificity, and LOD values as low as 100 fM were determined.

A similar study was performed by Kocabey et al. in 2023 [134]. Here, the assay was implemented on a linear, 8-helix-bundle (HB) DNA origami, allowing multiplexing of up to four miRNAs by analyzing the distance of the detected signal from two boundary markers on one edge of the structure (Figure 8C). Efficient target capture was ensured by incorporating four capture probes per target miRNA. Furthermore, an additional target labeling step using bridge oligonucleotides was implemented. These bridge sequences consisted of a target-specific part and a universal 8-nt DNA-PAINT docking site, enabling detection of different miRNA targets using the same DNA-PAINT imager sequence. Excellent specificity was demonstrated, and an LOD value of 11 fM was calculated for miR-153. Also here, detection in complex matrices was shown, by successfully validating the system for miRNA detection in cancer cell line RNA extracts and in plasma samples from cancer patients and healthy donors. The same authors finally extended their work towards POC applications by showing origami-enabled SM Ebola RNA detection using DNA-PAINT on a smartphone-based microscope (Figure 8D) [135]. To overcome the limited resolution of this readout device compared to lab-based microscopes, DNA origami dimers were prepared to obtain an increased distance between the boundary markers and the capture probes.

These DNA-PAINT-based approaches offer highly multiplexed readout through precise spatial encoding on DNA origami nanostructures. As such, the technique may be particularly valuable for detection of complex biomarker panels. The examples shown here all have an application-oriented perspective, exemplified by low target concentrations and detection in complex biological sample matrices (Table 2). Recent advances further support application-readiness by using universal imager strands and assay demonstration on simplified, smartphone-based microscopes.

### 4.2. Dynamic DNA Nanostructures

Using dynamic DNA origami structures, binding of (small) target analytes can be translated into large conformational changes. These nanostructures typically have a hinge-like geometry that switches between an open and closed state upon target detection. The readout of such dynamic DNA nanodevices is often based FRET, which relies on the non-radiative energy transfer between a donor and an acceptor fluorophore. When donor and acceptor are in close proximity, donor excitation results in acceptor emission. As the efficiency of this energy transfer process is inversely proportional to the 6th power of the distance between the dyes, FRET is a highly sensitive method to visualize conformational changes [145]. Since 2009, such concepts have been explored with the development of dynamic DNA boxes where opening and closing of the lid could be controlled with DNA or protein inputs [146,147,148,149]. However, while these concepts are theoretically SM-sensitive, they were analyzed with ensemble FRET measurements instead of SM-FRET. However, they still represent an important predecessor towards DNA origami-based SM-FRET sensors.

**Figure 9:**
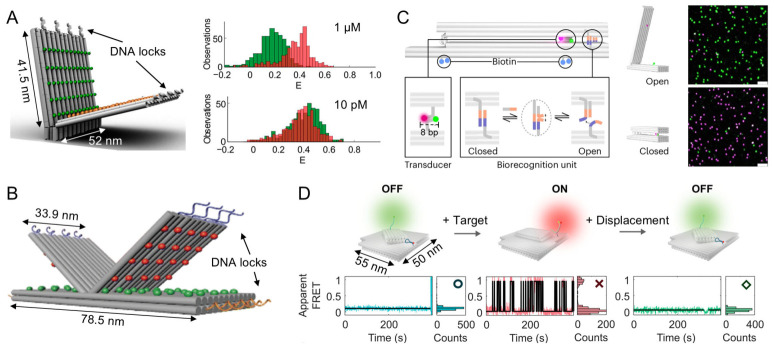
DNA origami-enabled SM detection with fluorescence as a readout using dynamic DNA nanostructures. (**A**) An optimized number of 48 FRET donor-acceptor pairs is attached to two halves of a dynamic DNA nanostructure, enabling analysis with a standard fluorescence microscope. The structure is closed with four dsDNA locks and can be opened by a complementary ssDNA target in a concentration-dependent manner. Scale bars are 2 µm. Adapted with permission from Ref. [136], © 2018 the American Chemical Society. (**B**) By implementing two lids on a flat base, a multiplex concept is developed. Each lid is closed by four dsDNA locks and can be opened by its respective DNA or RNA target in a concentration-dependent manner. Adapted with permission from Ref. [137], originally published under CC BY-NC 3.0 license. (**C**) The hinge concept was further optimized towards a modular, tunable, and widely applicable sensing approach using a different structure (~100 nm length). A robust signal is ensured by attaching the FRET pair on two 8 bp complementary ssDNA sequences. The versatility of the concept was demonstrated by tuning the response window with various approaches and by implementing multiple assays for NA as well as non-NA targets. Adapted with permission from Ref. [138], © 2024 Springer Nature. (**D**) A rotating FRET nanodevice was proposed as an alternative geometry. The device switches to its ON state when target NA is added, indicated by an increased FRET signal. The OFF state can be restored with a strand displacement mechanism. A multiplex version of the device was also developed employing two readout methods: FRET and fluorescence quenching. Adapted with permission from Ref. [139], © 2026 the American Chemical Society.

The first DNA origami hinge structure for SM detection with FRET-based readout was reported in 2018 by Selnihhin et al. Their aim was to design a DNA nanostructure capable of generating a signal high enough to be detected with a standard fluorescence microscope. To achieve this, a structure was designed consisting of two DNA origami plates, each carrying an optimized total of 48 donor or acceptor fluorophores, connected at a common foot (Figure 9A). The structure was locked in its closed state by four dsDNA locks, resulting in high FRET in absence of target DNA. Addition of the target DNA opened the structures and reduced the FRET signal. Excellent specificity was shown (i.e., no opening by non-target DNA) and by transitioning from ensemble to SM measurements, the LOD could be improved from 2 nM to 100 pM [136]. In 2022, this concept was extended to multiplex detection of cancer biomarkers by Domljanovic et al. Their structure consisted of one flat base, with two lids connected at the center of the base (Figure 9B). Each half of this dynamic DNA nanostructure could then be opened by their respective target via interaction with four dsDNA locks. They demonstrated this concept with a FRET-based readout and with a fluorophore-quencher (FQ)-based readout, where opening of the sensor resulted in a fluorescence increase. This concept was used for single- and multiplex detection of DNA analogs of miRNA, synthetic miRNA, and miRNA extracted from MCF-7 breast cancer cells, reaching LOD values as low as 1 pM [137].

The most versatile version of a DNA origami FRET-based SM biosensor to date was introduced in 2024 by Grabenhorst et al. [138]. Their structure comprised two rigid DNA arms connected at a central hinge, with each arm carrying a donor or acceptor fluorophore on an ssDNA staple extension (Figure 9C). With a weak 8 bp hybridization interaction between these extensions, a robust FRET signal was ensured when the structure was closed. This structure was employed to develop a modular, tunable, and widely applicable sensing approach by ensuring (1) strong signal generation upon target detection, and (2) flexibility in the type and number of bioreceptors implemented, allowing adaptation to the selected application and tuning of the corresponding response window to relevant target concentrations. This was first demonstrated with a 17-nt ssDNA model target, by incorporating a dsDNA lock with a toehold on the hinge. Using this model target, the authors demonstrated tuning of the response window with three strategies: (1) increasing the number of locks from two to four or six narrowed the response window and pushed it to higher target concentrations; (2) lowering the NaCl concentration from 400 mM to 50 mM shifted the response window to lower target concentrations due to increased electrostatic repulsion between the hinge arms; and (3) implementing a weak, 5 bp transient DNA–DNA interaction as an additional lock pushed the response window to higher target concentrations. Furthermore, the multivalency approach successfully provided discrimination between the ssDNA target and single-mismatch sequences. While demonstrated here with a model target, these tuning approaches are generally applicable to DNA origami hinges and therefore represent a toolbox for designing FRET-based DNA origami SM sensors for a wide variety of clinically relevant target analytes.

This versatility was demonstrated by extending to non-NA targets (Figure 9C). First, anti-digoxigenin (anti-Dig) antibodies could be detected after implementing two Dig antigens on each hinge arm in addition to six dsDNA locks. In presence of the DNA key sequence, the structure remained closed if anti-Dig antibodies were also present, enabling anti-Dig detection down to 10 pM. This assay remained functional in 50% human plasma and was highly specific, showing no cross-reactivity with anti-dinitrophenyl (anti-DNP) antibodies. Next, with a similar assay concept, employing dsDNA locks combined with aptamer pairs on the hinge arms, picomolar concentrations (≥100 pM) of platelet-derived growth factor BB (PDGF-BB) were detected. Additionally, enzymatic activity of the Xhol restriction enzyme was detected by implementing four dsDNA locks with a Xhol restriction site. After a 10 min incubation with the enzyme, a substantial fraction of the hinge nanostructures had opened. Furthermore, RNA targets were detected with a clustered regularly interspaced short palindromic repeats (CRISPR) and CRISPR-associated protein 9 (Cas9)-based assay. Here, dsDNA locks were implemented and the key sequence was embedded in a DNA hairpin with a Cas9 recognition site. The target RNA, acting as the CRISPR-RNA (crRNA), activated the Cas9 ribonucleoprotein (RNP) complex, cleaving the DNA hairpin and releasing the key sequence, thereby opening the DNA hinges. Finally, multiplex assay formats were demonstrated for simultaneous detection of anti-Dig and anti-DNP antibodies, as well as Xhol nuclease and an ssDNA target [123].

Finally, stepping away from the hinge geometry, Tsang et al. demonstrated a rotating DNA nanodevice for multiplex and continuous (reversible) detection of NA targets in 2026 [139]. Here, a 14 HB arm and a 2-layer flat base were folded from the same scaffold, resulting in a structure that allows the arm to rotate independently of the base. This structure was turned into an NA sensor by implementing two fluorophore-modified staple extensions: a Cy3-modified hairpin on the arm, and a Cy5-modified capture staple on the base. In absence of target NA, this structure prefers its OFF state, where the hairpin is closed and no FRET is observed (Figure 9D). Presence of the target NA opens the hairpin, switching the structure to its ON state, resulting in an increased FRET signal. By introduction of a displacement strand, the interaction can be reversed, switching the structure back to its OFF state. A multiplex detection concept was also demonstrated (in bulk) by employing a dual detection mechanism based on FRET for one target and fluorescence quenching for the second target.

While the dynamic hinge-like DNA nanostructures were first introduced as a general DNA origami design concept [150], further research always focused on SM detection in a diagnostic context, and demonstrating assay performance in clinically relevant sample matrices (Table 2). This illustrates that in recent years, there has been a shift in focus from proof-of-concept SM detection concepts towards application-oriented studies. With recent examples showing a great degree of modularity and adaptability to different targets and assay conditions the approach holds great promise towards real-life applicability.

### 4.3. Fluorescent Nanoantennas

As described for SM-SERS (see Section 3.3), optical signals can be amplified with nanoantennas, exploiting LSPRs in the plasmonic hotspot between two MNPs. SM-fluorescence measurements typically yield ON/OFF signals that are significantly easier to interpret than the complex molecular fingerprints obtained with SM-SERS, and, hence, are often more practical for SM detection based applications. In this context, numerous studies have reported DNA origami-based nanoantennas for SM fluorescence amplification. One particular goal in these studies is the application of nanoantennas for fluorescence enhancement to enable SM detection on simple imaging setups [142,143,144]. Interestingly, whereas SM-SERS simply requires the target molecule to be placed as close to the MNPs as possible, this is not the case for fluorescent dyes, as fluorescence quenching can occur [151], thus requiring optimal and precise placement of the dye within the interparticle gap, which can be achieved with DNA origami [152].

**Figure 10:**
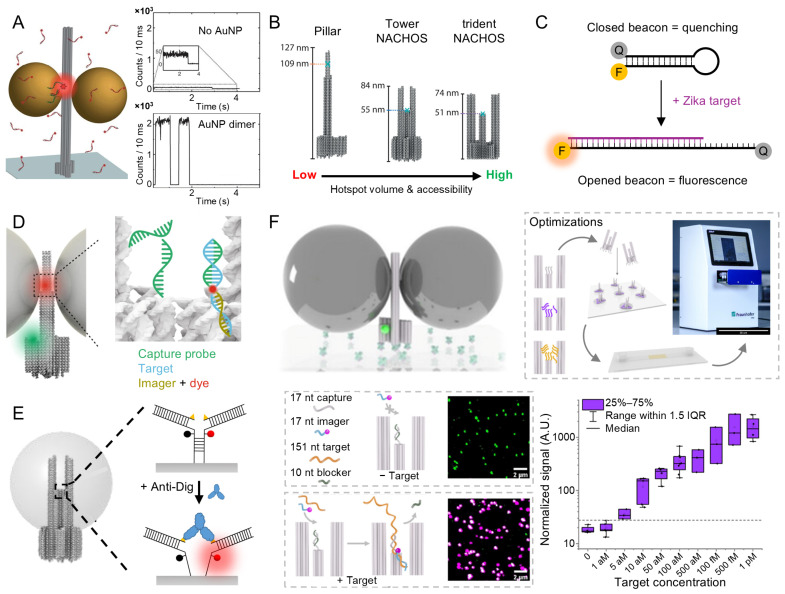
DNA origami-enabled SM detection with fluorescence as a readout using optical nanoantennas. (**A**) A DNA origami pillar nanostructure carrying two AuNPs results in up to 117-fold fluorescence amplification. Adapted with permission from Ref. [140], © 2012 The American Association for the Advancement of Science. (**B**) Different DNA nanoantenna designs were developed through the years, from pillar nanostructures to trident NACHOS. Later versions of the design aimed at increasing the volume and accessibility of the hotspot. Adapted with permission from Ref. [156], originally published under CC BY-NC-ND 4.0 license. (**C**) The pillar DNA nanoantenna was used for detection of Zika virus NA sequences. An FQ-labeled molecular beacon was immobilized at the hotspot. Opening upon target NA hybridization resulted in a fluorescence response by increasing the FQ distance. (**D**) The tower NACHOS DNA nanostructure was used for detection of NA-specific to antibiotic-resistant *K. pneumoniae* in a sandwich assay. Adapted from Ref. [142], originally published under CC BY 4.0 license. (**E**) The same structure was equipped with a molecular beacon for detection of anti-Dig antibodies. Adapted with permission from Ref. [143], originally published under CC BY-NC-ND 4.0 license. (**F**) The trident NACHOS DNA nanostructure was optimized by addition of a blocker sequence, an increased number of capture probes, nanoantenna immobilization on nanoarrays in a microfluidic chip, and development of an in-house fluorescence reader. NA targets related to antibiotic-resistant *K. pneumoniae* were detected down to a LOD of ~5 aM. Scale bars are 2 µm. Adapted from Ref. [144], originally published under CC BY 4.0 license.

The lab of Philip Tinnefeld pioneered this field in 2012 with a nanoantenna design comprising a 220 nm DNA pillar nanostructure consisting of a 12 HB DNA origami, with a foot of three 6 HBs (Figure 10A) [140]. Fluorescence enhancement of ATTO647N was shown by attaching one or two AuNPs to this pillar, resulting in a maximum fluorescence enhancement of 117-fold for a single dye in the 23 nm gap between two 100 nm AuNPs. With this structure, the authors demonstrated a basic SM ssDNA target detection concept by visualizing the transient binding of a ATTO655-labeled sequence, as well as SM observations of conformational dynamics in a Holliday junction. Later, with an improved version of the nanoantenna design, the same authors demonstrated up to 5000-fold fluorescence enhancement [153].

After their seminal work, the Tinnefeld lab remained one of the main innovators in the DNA nanoantenna field, further optimizing the concept as extensively described in their review from 2021 [154]. Several key improvements were implemented. First, the interparticle gap was reduced by narrowing the structure to a 6 HB at the MNP attachment site, increasing fluorescence amplification [153]. Second, the implementation of large MNPs (≥80 nm) was optimized [152] and AgNPs were implemented instead of AuNPs, extending the usable spectral range by outperforming AuNP-based nanoantennas in the green and blue wavelengths, while performing similarly in the red [155]. Third, further modifications to the DNA nanostructure were made to increase the volume and accessibility of the hotspot while maintaining a small interparticle gap, resulting in the (trident) NanoAntenna with Cleared HOtSpot (NACHOS) DNA nanostructure (Figure 10B) [142,156]. Whereas the initial pillar design could only host a single dye-labeled staple, effectively having a near-zero hotspot volume, the final version of the NACHOS DNA origami, trident NACHOS, increased the hotspot volume to 7 zL (19 nm × 368.9 nm^2^), enabling SM detection of diverse analytes [156].

Throughout their development, the different versions of the nanoantenna design were used in various SM detection concepts. The earliest example in 2017 targeted viral NAs originating from the Zika virus [141]. An FQ-labeled (ATTO 647N and BlackBerry Quencher 650) molecular beacon was attached at the hotspot of a pillar nanoantenna bearing only one AgNP, to avoid steric inhibition of target binding (Figure 10C). The beacon contained a region complementary to a synthetic, 23-nt long ssDNA analog of Zika virus RNA. Upon target hybridization, the molecular beacon opened, increasing the FQ distance, yielding a nanoantenna-amplified fluorescence response. The authors further demonstrated discrimination between targets with up to three mismatches, successful detection in heat-inactivated human serum and detection of Zika virus RNA. Multiplex detection of two ssDNA targets was enabled by color-barcoding the nanoantennas with a fluorescent label at their base, allowing discrimination between different nanoantennas, while keeping the same labels in the molecular beacon. Despite these advances, two important limitations remained. First, only limited fluorescence enhancement was observed, due to the single AgNP, highlighting the limitations of the pillar design in terms of hotspot accessibility [154]. Second, this type of assay suffers from an increased number of false positives in the amplified system, compared to the non-amplified system, because dark quenchers show faster bleaching when placed in a plasmonic hotspot [157].

In 2021, the use of a tower NACHOS DNA origami for SM detection of ssDNA targets specific to antibiotic-resistant *K. pneumoniae* was reported, overcoming limitations related to hotspot accessibility [142]. Here, the hotspot between two 100 nm AgNPs was functionalized with three 17 nt long capture probes complementary to a 34 nt long DNA sequence originating from the *OXA-48* gene. Target detection was visualized with an Alexa Fluor 647-labeled detection probe, complementary to the remaining 17 nt of the target and overcoming earlier issues related to quencher photobleaching (Figure 10D). The nanoantenna enabled up to 461-fold fluorescence amplification, and target detection was demonstrated in buffer as well as in heat-inactivated human serum. Moreover, targets with ≤3 nts mismatch were discriminated and target detection (in buffer and serum) was shown on an in-house-developed, smartphone-based fluorescence microscope, highlighting the potential for point-of-care (POC) diagnostic applications. The same nanoantenna was also used for SM detection of anti-Dig antibodies, serving as a demonstrator case for larger, non-NA targets [143]. In this assay, an FQ (ATTO 647N and BlackBerry Quencher 650) DNA nanoswitch containing two Dig-labeled sequences opened upon anti-Dig binding, increasing FQ spacing and producing a fluorescent signal (Figure 10E). With this switch incorporated into the nanoantenna, anti-Dig antibodies were successfully detected down to a concentration of ~1 nM, with up to 60-fold signal amplification. The measurement was also successfully repeated with the smartphone-based microscope. However, this assay for larger non-NA targets shared the same limitations as before: only a single AgNP was used to accommodate the larger target size, limiting signal amplification, and false positives were observed due to photobleaching of the quencher.

In their most recent work, published in 2025, the authors pushed the nanoantenna concept towards attomolar LODs (Figure 10F) [144]. A sandwich assay, similar to what was reported earlier [142] for SM detection of a 151 nt long sequence originating from antibiotic-resistant *K. pneumoniae*, was implemented in the trident NACHOS structure and optimized towards ultrasensitive POC diagnostics by (1) implementing up to 10 capture strands in the hotspot, increasing the avidity at the binding site, (2) adding a short blocker strand complementary to the capture probe, reducing the number of false positives, (3) immobilizing the nanoantennas on nanopatterned surfaces to obtain a high surface density, (4) developing a simple benchtop fluorescence reader capable of analyzing large (3 mm × 2.5 mm) fields of view, enhancing the sensitivity through increased probability of detecting rare target-binding events, and (5) incorporation into a microfluidic chip to enhance binding kinetics. Using this optimized assay, an LOD of ~5 aM was obtained, which is the lowest LOD reported across all DNA origami-enabled SM detection concepts in this review (Table 1 and Table 2). Furthermore, silicification, forming a 3 nm silica shell on the DNA origami, was explored to improve the stability of the DNA nanoantennas in complex sample media and to stabilize their attachment on the surface of the chip. With silicified nanoantennas, the authors still reached an excellent LOD of ~10 aM in blood plasma. Finally, an overnight strand displacement procedure enabled chip reuse by near-complete removal of bound targets from the nanoantennas and restoring comparable signals upon re-incubation with 500 pM target [144].

From an application perspective, DNA origami-based fluorescence nanoantennas are among the most advanced SM sensing approaches, demonstrating LODs that are unmatched by any other DNA origami-based SM detection method (Table 2). One of their main advantages lies in the combination of a strongly amplified ON/OFF signal with a relatively simple optical readout, which was even demonstrated on simple readout setups. Similarly to the other examples with a fluorescent readout, these studies also show a stronger application orientation compared to the non-fluorescence-based approaches discussed earlier. As such, this indicates a remarkable maturation of the approach toward DNA origami-enabled SM detection that has taken place over the last ten years.

## 5. Conclusions and Future Directions

As shown by the many examples discussed above, DNA origami offers a versatile toolbox for the development of SM detection concepts. Clearly, the field has significantly evolved from the initial AFM concepts, first reported in 2008, to the nanoantennas with attomolar sensitivity reported more recently. In the early days, SM detection was rather a “side-effect” of the employed read-out methodology, often AFM, which is inherently SM, in studies aiming to show a proof-of-concept target detection mechanism. In more recent publications, robust SM detection has become the explicit goal, as evidenced by the increased reporting of LOD values and detection in complex matrices (Table 1 and Table 2). Additionally, while early work relied on AFM as the primary read-out method, AFM has since shifted to being mainly a characterization technique. Even today, most of the studies on dynamic DNA nanostructures for SM detection still employ AFM imaging or other direct visualization techniques like transmission electron microscopy (TEM) as an intermediate step to show target-induced opening or closing of the nanostructure (e.g., [54,55,57]). Notably, the most sensitive concepts were all reported recently (after 2018) and mainly rely on fluorescence, which is especially well-suited as a readout method for SM applications. This further indicates that the observed trend is a recent phenomenon.

Practical applicability of the platforms discussed in this review is inevitably influenced by the characteristics of the proposed readout mechanism. AFM enables direct SM visualization of nanostructure–target interactions with unmatched spatial resolution, but is limited by low throughput and the need for specialized instrumentation, restricting its use mainly to characterization. Nanopore-based methods provide an inherently digital, label-free readout with relatively high throughput and are supported by well-established technological concepts, making them more readily applicable. However, signal interpretation in a practical context may remain challenging, especially for non-experts. Origami-based SM-SERS nanoantennas offer chemically specific detection through distinct molecular fingerprints, but their implementation is complicated by the need for precise hotspot design and rigorous data analysis. In contrast, fluorescence-based approaches generally produce simpler ON/OFF signals, facilitating higher throughput and straightforward multiplexing. As such, while no single platform is universally superior, fluorescence-based systems currently appear to be the most advanced for practical SM sensing applications. Meanwhile, AFM, nanopores, and SM-SERS offer complementary advantages for sensor development in more specialized analytical contexts.

The field has also progressed significantly in terms of applications. Whereas early concepts often demonstrated detection of a model target (e.g., thrombin or streptavidin) in buffer, recent studies increasingly report the detection of clinically relevant target molecules, including demonstration in clinically relevant sample matrices. Nevertheless, proof-of-concept clinical studies remain limited, with only one example published so far, detecting miRNA in plasma samples from cancer patients [134]. As such, there are still quite a few steps to be taken towards real-life applicability. Part of the solution to this challenge lies in a more thorough understanding of how DNA origami is affected by physiological sample media and how to overcome potential issues due to interactions with such media, which is a topic extensively studied in the DNA origami field [158].

As the field continues to advance, future developments will likely focus on SM detection of non-NA targets. While recent studies demonstrate attomolar detection of NA targets [144] and versatile detection of non-NA targets [138], these two achievements have not yet been combined. Although the focus on NA targets is partly fueled by the inherent DNA-based nature of the detection concepts, proteins remain important disease biomarkers that could benefit from SM detection [159,160]. In combination with DNA origami, aptamers are often referred to as the ideal bioreceptor for protein targets, due to their NA nature allowing straightforward implementation. However, high-performance aptamers for clinically relevant targets remain scarce [161] and the field is heavily dominated by thrombin as a model target [162]. In this context, the continuously expanding toolbox of DNA origami functionalization methods will likely be critical for developing DNA origami-based SM detection concepts with robustly attached protein bioreceptors [31]. Overall, we expect continued efforts toward improving assay performance, which, as demonstrated by recent work on DNA origami nanoantennas, requires careful optimization of both the sensing architecture and the overall assay design [144].

At the same time, it remains important to distinguish between approaches that enable individual nanostructure–target interrogation and those that provide integrated SM detection assays. In the broader definition adopted in this review, both are considered SM detection, although the degree of analytical integration varies across implementations, with implications for real-world and POC applicability. While clear progress has been made, many concepts remain at the proof-of-concept stage, and further advances in assay integration, robustness, and scalability are needed before routine clinical implementation can be achieved. Nevertheless, building on recent innovations, DNA origami-based SM detection approaches are moving toward practical, ultrasensitive detection of clinically relevant biomarkers. Several studies have already demonstrated routes toward POC implementation through cost-efficient and even smartphone-based readout technologies [135,142,143,144], reducing the need for complex instrumentation typically associated with SM detection. Integration with micro- and nanopatterning technologies, as well as microfluidics, could further improve robustness and user-friendliness for real-life applications [144,163]. In parallel, the underlying design principles have also been translated into simpler assay concepts (e.g., lateral flow), which may provide practical solutions in scenarios requiring high sensitivity, though not necessarily SM resolution [164]. Despite these advances, challenges related to scalability and cost-effective implementation remain to be addressed for widespread adoption.

While this review aimed at summarizing key trends grouped in a few overarching categories, other notable examples of origami-enabled SM detection exist. These include a target-responsive dynamic nanostructure, visualized with TEM [165], a nanoantenna based on whispering gallery mode sensing [166], a mechanochemical sensor measuring target-induced displacement of two optically trapped beads attached to a DNA nanostructure [167], a FRET-based sensor for vesicle detection [168], and a bead-based digital assay employing DNA origami to precisely control aptamer placement [169], among others. Moreover, there are also a wide variety of DNA origami concepts that are inherently SM, i.e., a DNA nanostructure carrying one (or a few) bioreceptor(s), but that do not reach SM sensitivity due to the employed read-out methodology, such as a recent electrochemical sandwich assay, where a DNA origami was decorated with up to 70 electrochemical reporters to induce an amplified response [170]. Similarly, single target-triggered, plasmonic strategies using circular dichroism-based readouts in bulk have also been demonstrated for NAs [171,172,173] and small molecules [174]. Furthermore, the field of SM biophysics enables a whole range of applications beyond SM detection, with SM analysis studies performed with various concepts discussed in this review, including AFM with direct observation [175,176,177,178,179], DNA origami-modified nanopores [88,180,181,182,183,184], dynamic hinge-like nanostructures [185,186,187,188,189,190,191], and nanoantennas with fluorescence readout [154]. Beyond these examples, DNA origami has also enabled SM detection and biophysics measurements through alternative transduction mechanisms, including electrochemical nanoimpact detection [192] and force- or tension-based nanosensors [193,194,195]. These approaches further illustrate the broad applicability of DNA origami as a platform for SM studies across diverse readout modalities. Overall, the rapid progress achieved over the past decade suggests that DNA origami-enabled SM detection is transitioning from a primarily proof-of-concept technology toward a practical sensing platform. While current efforts remain largely focused on diagnostic applications, the technology holds promise for broader use in other areas, including drug discovery, food safety, and environmental monitoring [196,197].

## Data Availability

No new data were created in this study.

## Abbreviations

The following abbreviations are used in this manuscript:

SM	Single-molecule
LOD	Limit of detection
NA	Nucleic acid
2D	Two-dimensional
3D	Three-dimensional
ss	Single-stranded
nt	Nucleotide
ds	Double-stranded
FRET	Förster resonance energy transfer
MNP	Metal nanoparticle
AFM	Atomic force microscopy
SERS	Surface-enhanced Raman scattering
RT-PCR	Reverse-transcription PCR
ATP	Adenosine triphosphate
FAM	Carboxyfluorescein
IgG	Immunoglobulin G
miRNA	Micro RNA
GTP	Guanosine triphosphate
SSN	Solid-state nanopore
SiN	Silicon-nitride
bp	Basepair
holo-hSTf	Holo human serum transferrin
CTP	Cytidine triphosphate
CRP	C-reactive protein
LSPRs	Localized surface plasmon resonances
AuNP	Gold nanoparticle
AgNP	Silver nanoparticle
BSA	Bovine serum albumin
cyt c	Cytochrome c
HRP	Horseradish peroxidase
SHRImP	SM high-resolution imaging with photobleaching
dSTORM	Direct stochastic optical reconstruction microscopy
DNA-PAINT	DNA points accumulation for imaging in nanoscale topography
qPAINT	Quantitative PAINT
RESI	Resolution enhancement by sequential imaging
HB	Helix bundle
FQ	Fluorophore-quencher
Dig	Digoxigenin
DNP	Dinitrophenyl
PDGF-BB	Platelet-derived growth factor BB
CRISPR	Clustered regularly interspaced short palindromic repeats
Cas9	CRISPR-associated protein 9
crRNA	CRISPR-RNA
RNP	Ribonucleoprotein
NACHOS	Nanoantenna with cleared hotspot
POC	Point-of-care
TEM	Transmission electron microscopy

## References

[1] Walt D.R. (2013). Optical Methods for Single Molecule Detection and Analysis. Anal. Chem..

[2] Kan C.W., Tobos C.I., Rissin D.M., Wiener A.D., Meyer R.E., Svancara D.M., Comperchio A., Warwick C., Millington R., Collier N. (2020). Digital Enzyme-Linked Immunosorbent Assays with Sub-Attomolar Detection Limits Based on Low Numbers of Capture Beads Combined with High Efficiency Bead Analysis. Lab Chip.

[3] Zhang Y., Gu H., Xu H. (2024). Recent Progress in Digital Immunoassay: How to Achieve Ultrasensitive, Multiplex and Clinical Accessible Detection?. Sens. Diagn..

[4] Moerner W.E. (2002). A Dozen Years of Single-Molecule Spectroscopy in Physics, Chemistry, and Biophysics. J. Phys. Chem. B.

[5] Rothemund P.W.K. (2006). Folding DNA to Create Nanoscale Shapes and Patterns. Nature.

[6] Xu A., Harb J.N., Kostiainen M.A., Hughes W.L., Woolley A.T., Liu H., Gopinath A. (2017). DNA Origami: The Bridge from Bottom to Top. MRS Bull..

[7] Rajendran A., Endo M., Sugiyama H. (2012). Single-Molecule Analysis Using DNA Origami. Angew. Chem. Int. Ed..

[8] Wang S., Zhou Z., Ma N., Yang S., Li K., Teng C., Ke Y., Tian Y. (2020). DNA Origami-Enabled Biosensors. Sensors.

[9] Dass M., Gür F.N., Kołątaj K., Urban M.J., Liedl T. (2021). DNA Origami-Enabled Plasmonic Sensing. J. Phys. Chem. C.

[10] Hao Z., Kong L., Ruan L., Deng Z. (2024). Recent Advances in DNA Origami-Enabled Optical Biosensors for Multi-Scenario Application. Nanomaterials.

[11] Kogikoski S., Gonçalves Pontes R., Paschoalino W.J., Kubota L.T. (2025). Opportunities for DNA Origami in Electrochemical Sensing Applications. ACS Electrochem..

[12] Douglas S.M., Marblestone A.H., Teerapittayanon S., Vazquez A., Church G.M., Shih W.M. (2009). Rapid Prototyping of 3D DNA-Origami Shapes with CaDNAno. Nucleic Acids Res..

[13] Castro C.E., Kilchherr F., Kim D.-N., Shiao E.L., Wauer T., Wortmann P., Bathe M., Dietz H. (2011). A Primer to Scaffolded DNA Origami. Nat. Methods.

[14] Wagenbauer K.F., Engelhardt F.A.S., Stahl E., Hechtl V.K., Stömmer P., Seebacher F., Meregalli L., Ketterer P., Gerling T., Dietz H. (2017). How We Make DNA Origami. ChemBioChem.

[15] Doty D., Lee B.L., Stérin T. (2020). Scadnano: A Browser-Based, Scriptable Tool for Designing DNA Nanostructures. Proceedings of the DNA 2020: Proceedings of the 26th International Conference on DNA Computing and Molecular Programming.

[16] Levy N., Schabanel N. (2021). Ensnano: A 3d Modeling Software for Dna Nanostructures. Proceedings of the DNA 2021: Proceedings of the 27th International Conference on DNA Computing and Molecular Programming.

[17] Pfeifer W.G., Huang C.-M., Poirier M.G., Arya G., Castro C.E. (2023). Versatile Computer-Aided Design of Free-Form DNA Nanostructures and Assemblies. Sci. Adv..

[18] Veneziano R., Ratanalert S., Zhang K., Zhang F., Yan H., Chiu W., Bathe M. (2016). Designer Nanoscale DNA Assemblies Programmed from the Top Down. Science.

[19] Jun H., Zhang F., Shepherd T., Ratanalert S., Qi X., Yan H., Bathe M. (2019). Autonomously Designed Free-Form 2D DNA Origami. Sci. Adv..

[20] Chen X., Wang Q., Peng J., Long Q., Yu H., Li Z. (2018). Self-Assembly of Large DNA Origami with Custom-Designed Scaffolds. ACS Appl. Mater. Interfaces.

[21] Engelhardt F.A.S., Praetorius F., Wachauf C.H., Brüggenthies G., Kohler F., Kick B., Kadletz K.L., Pham P.N., Behler K.L., Gerling T. (2019). Custom-Size, Functional, and Durable DNA Origami with Design-Specific Scaffolds. ACS Nano.

[22] Wu H., Zhang T., Qin Y., Xia X., Bai T., Gu H., Wei B. (2024). Expanding DNA Origami Design Freedom with De Novo Synthesized Scaffolds. J. Am. Chem. Soc..

[23] Nickels P.C., Ke Y., Jungmann R., Smith D.M., Leichsenring M., Shih W.M., Liedl T., Högberg B. (2014). DNA Origami Structures Directly Assembled from Intact Bacteriophages. Small.

[24] Marchi A.N., Saaem I., Vogen B.N., Brown S., LaBean T.H. (2014). Toward Larger DNA Origami. Nano Lett..

[25] Zhan P., Peil A., Jiang Q., Wang D., Mousavi S., Xiong Q., Shen Q., Shang Y., Ding B., Lin C. (2023). Recent Advances in DNA Origami-Engineered Nanomaterials and Applications. Chem. Rev..

[26] Castro C.E., Su H.-J., Marras A.E., Zhou L., Johnson J. (2015). Mechanical Design of DNA Nanostructures. Nanoscale.

[27] Ke Y., Castro C., Choi J.H. (2018). Structural DNA Nanotechnology: Artificial Nanostructures for Biomedical Research. Annu. Rev. Biomed. Eng..

[28] Li R., Madhvacharyula A.S., Du Y., Adepu H.K., Choi J.H. (2023). Mechanics of Dynamic and Deformable DNA Nanostructures. Chem. Sci..

[29] Shaw A., Benson E., Högberg B. (2015). Purification of Functionalized DNA Origami Nanostructures. ACS Nano.

[30] Madsen M., Gothelf K.V. (2019). Chemistries for DNA Nanotechnology. Chem. Rev..

[31] Knappe G.A., Wamhoff E.-C., Bathe M. (2022). Functionalizing DNA Origami to Investigate and Interact with Biological Systems. Nat. Rev. Mater..

[32] Wang Y.-C., Wang Y.-Y., Wu J., Xu P., Cui H.-B., Guo P., Cao H., Chen W., Guan G., Han M.-Y. (2026). Chemical and Enzymatic Strategies for the Synthesis, Ligation, Assembly and Emerging Applications of DNA Nanostructures. Biomater. Sci..

[33] Rinker S., Ke Y., Liu Y., Chhabra R., Yan H. (2008). Self-Assembled DNA Nanostructures for Distance-Dependent Multivalent Ligand–Protein Binding. Nat. Nanotechnol..

[34] Saccà B., Meyer R., Erkelenz M., Kiko K., Arndt A., Schroeder H., Rabe K.S., Niemeyer C.M. (2010). Orthogonal Protein Decoration of DNA Origami. Angew. Chem. Int. Ed..

[35] Sharma J., Chhabra R., Andersen C.S., Gothelf K.V., Yan H., Liu Y. (2008). Toward Reliable Gold Nanoparticle Patterning On Self-Assembled DNA Nanoscaffold. J. Am. Chem. Soc..

[36] Steinhauer C., Jungmann R., Sobey T.L., Simmel F.C., Tinnefeld P. (2009). DNA Origami as a Nanoscopic Ruler for Super-Resolution Microscopy. Angew. Chem. Int. Ed..

[37] Curtin K., Fike B.J., Binkley B., Godary T., Li P. (2022). Recent Advances in Digital Biosensing Technology. Biosensors.

[38] Lyubchenko Y.L., Shlyakhtenko L.S., Ando T. (2011). Imaging of Nucleic Acids with Atomic Force Microscopy. Methods.

[39] Main K.H.S., Provan J.I., Haynes P.J., Wells G., Hartley J.A., Pyne A.L.B. (2021). Atomic Force Microscopy—A Tool for Structural and Translational DNA Research. APL Bioeng..

[40] Hoogenboom B.W. (2021). Stretching the Resolution Limit of Atomic Force Microscopy. Nat. Struct. Mol. Biol..

[41] Ke Y., Lindsay S., Chang Y., Liu Y., Yan H. (2008). Self-Assembled Water-Soluble Nucleic Acid Probe Tiles for Label-Free RNA Hybridization Assays. Science.

[42] Ke Y., Nangreave J., Yan H., Lindsay S., Liu Y. (2008). Developing DNA Tiles for Oligonucleotide Hybridization Assay with Higher Accuracy and Efficiency. Chem. Commun..

[43] Numajiri K., Kimura M., Kuzuya A., Komiyama M. (2010). Stepwise and Reversible Nanopatterning of Proteins on a DNA Origami Scaffold. Chem. Commun..

[44] Xi C., Zhang J., Song L., Zhang Y., Wang S., Li M., Wang F., Fan C., Wang H., Zuo X. (2026). DNA Framework-Encoded Digital Recorder for Bacterial Discrimination. ACS Nano.

[45] Kuzuya A., Sakai Y., Yamazaki T., Xu Y., Komiyama M. (2011). Nanomechanical DNA Origami “single-Molecule Beacons” Directly Imaged by Atomic Force Microscopy. Nat. Commun..

[46] Bell N.A.W., Engst C.R., Ablay M., Divitini G., Ducati C., Liedl T., Keyser U.F. (2012). DNA Origami Nanopores. Nano Lett..

[47] Wei R., Martin T.G., Rant U., Dietz H. (2012). DNA Origami Gatekeepers for Solid-State Nanopores. Angew. Chem. Int. Ed..

[48] Joty K., Ghimire M.L., Kahn J.S., Lee S., Alexandrakis G., Kim M.J. (2024). DNA Origami Incorporated into Solid-State Nanopores Enables Enhanced Sensitivity for Precise Analysis of Protein Translocations. Anal. Chem..

[49] Hernández-Ainsa S., Bell N.A.W., Thacker V.V., Göpfrich K., Misiunas K., Fuentes-Perez M.E., Moreno-Herrero F., Keyser U.F. (2013). DNA Origami Nanopores for Controlling DNA Translocation. ACS Nano.

[50] Raveendran M., Lee A.J., Sharma R., Wälti C., Actis P. (2020). Rational Design of DNA Nanostructures for Single Molecule Biosensing. Nat. Commun..

[51] Ding T., Yang J., Wang J., Pan V., Lu Z., Ke Y., Zhang C. (2022). Shaped DNA Origami Carrier Nanopore Translocation Influenced by Aptamer Based Surface Modification. Biosens. Bioelectron..

[52] Long L., Johnson J.A., Ren R., Di Michele L., Edel J.B., Ivanov A.P. (2025). Reconfigurable DNA Origami Hinges for Nanopore Detection of MicroRNA. Nano Res..

[53] Chau C.C.C., Gupta V., Heath G.R., Wälti C., Actis P. (2026). Visualizing and Quantifying MicroRNA-Induced DNA Origami Separation at the Nanoscale. Angew. Chem. Int. Ed..

[54] Heck C., Kanehira Y., Kneipp J., Bald I. (2018). Placement of Single Proteins within the SERS Hot Spots of Self-Assembled Silver Nanolenses. Angew. Chem. Int. Ed..

[55] Tanwar S., Kaur V., Kaur G., Sen T. (2021). Broadband SERS Enhancement by DNA Origami Assembled Bimetallic Nanoantennas with Label-Free Single Protein Sensing. J. Phys. Chem. Lett..

[56] Kaur C., Kaur V., Rai S., Sharma M., Sen T. (2023). Selective Recognition of the Amyloid Marker Single Thioflavin T Using DNA Origami-Based Gold Nanobipyramid Nanoantennas. Nanoscale.

[57] Sharma M., Kaur C., Singhmar P., Rai S., Sen T. (2024). DNA Origami-Templated Gold Nanorod Dimer Nanoantennas: Enabling Addressable Optical Hotspots for Single Cancer Biomarker SERS Detection. Nanoscale.

[58] Tapio K., Mostafa A., Kanehira Y., Suma A., Dutta A., Bald I. (2021). A Versatile DNA Origami-Based Plasmonic Nanoantenna for Label-Free Single-Molecule Surface-Enhanced Raman Spectroscopy. ACS Nano.

[59] Schuknecht F., Kołątaj K., Steinberger M., Liedl T., Lohmueller T. (2023). Accessible Hotspots for Single-Protein SERS in DNA-Origami Assembled Gold Nanorod Dimers with Tip-to-Tip Alignment. Nat. Commun..

[60] Chhabra R., Sharma J., Ke Y., Liu Y., Rinker S., Lindsay S., Yan H. (2007). Spatially Addressable Multiprotein Nanoarrays Templated by Aptamer-Tagged DNA Nanoarchitectures. J. Am. Chem. Soc..

[61] Kuzuya A., Watanabe R., Yamanaka Y., Tamaki T., Kaino M., Ohya Y. (2014). Nanomechanical DNA Origami PH Sensors. Sensors.

[62] Kido S., Takahashi N., Miyazaki H., Kono S., Saito K., Kuzuya A. (2025). DNA Origami-Based Luminescent Biosensors Enabling Smartphone Detection of Nucleic Acid Sequences. ACS Omega.

[63] Dekker C. (2007). Solid-State Nanopores. Nat. Nanotechnol..

[64] Keyser U.F. (2011). Controlling Molecular Transport through Nanopores. J. R. Soc. Interface.

[65] Xue L., Yamazaki H., Ren R., Wanunu M., Ivanov A.P., Edel J.B. (2020). Solid-State Nanopore Sensors. Nat. Rev. Mater..

[66] Kumar Sharma R., Agrawal I., Dai L., Doyle P.S., Garaj S. (2019). Complex DNA Knots Detected with a Nanopore Sensor. Nat. Commun..

[67] Kasianowicz J.J., Brandin E., Branton D., Deamer D.W. (1996). Characterization of Individual Polynucleotide Molecules Using a Membrane Channel. Proc. Natl. Acad. Sci. USA.

[68] He Y., Tsutsui M., Zhou Y., Miao X.-S. (2021). Solid-State Nanopore Systems: From Materials to Applications. NPG Asia Mater..

[69] Keyser U.F. (2016). Enhancing Nanopore Sensing with DNA Nanotechnology. Nat. Nanotechnol..

[70] Ding T., Yang J., Pan V., Zhao N., Lu Z., Ke Y., Zhang C. (2020). DNA Nanotechnology Assisted Nanopore-Based Analysis. Nucleic Acids Res..

[71] Hernández-Ainsa S., Keyser U.F. (2014). DNA Origami Nanopores: Developments, Challenges and Perspectives. Nanoscale.

[72] Hall A.R., Scott A., Rotem D., Mehta K.K., Bayley H., Dekker C. (2010). Hybrid Pore Formation by Directed Insertion of α-Haemolysin into Solid-State Nanopores. Nat. Nanotechnol..

[73] Bell N.A.W., Thacker V.V., Hernández-Ainsa S., Fuentes-Perez M.E., Moreno-Herrero F., Liedl T., Keyser U.F. (2013). Multiplexed Ionic Current Sensing with Glass Nanopores. Lab Chip.

[74] Freedman K.J., Otto L.M., Ivanov A.P., Barik A., Oh S.-H., Edel J.B. (2016). Nanopore Sensing at Ultra-Low Concentrations Using Single-Molecule Dielectrophoretic Trapping. Nat. Commun..

[75] Chuah K., Wu Y., Vivekchand S.R.C., Gaus K., Reece P.J., Micolich A.P., Gooding J.J. (2019). Nanopore Blockade Sensors for Ultrasensitive Detection of Proteins in Complex Biological Samples. Nat. Commun..

[76] Wu Y., Yao Y., Cheong S., Tilley R.D., Gooding J.J. (2020). Selectively Detecting Attomolar Concentrations of Proteins Using Gold Lined Nanopores in a Nanopore Blockade Sensor. Chem. Sci..

[77] Plesa C., Kowalczyk S.W., Zinsmeester R., Grosberg A.Y., Rabin Y., Dekker C. (2013). Fast Translocation of Proteins through Solid State Nanopores. Nano Lett..

[78] Plesa C., van Loo N., Ketterer P., Dietz H., Dekker C. (2015). Velocity of DNA during Translocation through a Solid-State Nanopore. Nano Lett..

[79] Bell N.A.W., Keyser U.F. (2016). Digitally Encoded DNA Nanostructures for Multiplexed, Single-Molecule Protein Sensing with Nanopores. Nat. Nanotechnol..

[80] Bošković F., Zhu J., Chen K., Keyser U.F. (2019). Monitoring G-Quadruplex Formation with DNA Carriers and Solid-State Nanopores. Nano Lett..

[81] Chen K., Kong J., Zhu J., Ermann N., Predki P., Keyser U.F. (2019). Digital Data Storage Using DNA Nanostructures and Solid-State Nanopores. Nano Lett..

[82] Zhao X., Ma R., Hu Y., Chen X., Dou R., Liu K., Cui C., Liu H., Li Q., Pan D. (2019). Translocation of Tetrahedral DNA Nanostructures through a Solid-State Nanopore. Nanoscale.

[83] Bell N.A.W., Keyser U.F. (2015). Specific Protein Detection Using Designed DNA Carriers and Nanopores. J. Am. Chem. Soc..

[84] Plesa C., Ruitenberg J.W., Witteveen M.J., Dekker C. (2015). Detection of Individual Proteins Bound along DNA Using Solid-State Nanopores. Nano Lett..

[85] Yu J.-S., Lim M.-C., Huynh D.T.N., Kim H.-J., Kim H.-M., Kim Y.-R., Kim K.-B. (2015). Identifying the Location of a Single Protein along the DNA Strand Using Solid-State Nanopores. ACS Nano.

[86] Kong J., Bell N.A.W., Keyser U.F. (2016). Quantifying Nanomolar Protein Concentrations Using Designed DNA Carriers and Solid-State Nanopores. Nano Lett..

[87] Bošković F., Zhu J., Tivony R., Ohmann A., Chen K., Alawami M.F., Đorđević M., Ermann N., Pereira-Dias J., Fairhead M. (2023). Simultaneous Identification of Viruses and Viral Variants with Programmable DNA Nanobait. Nat. Nanotechnol..

[88] Plesa C., Ananth A.N., Linko V., Gülcher C., Katan A.J., Dietz H., Dekker C. (2014). Ionic Permeability and Mechanical Properties of DNA Origami Nanoplates on Solid-State Nanopores. ACS Nano.

[89] Alibakhshi M.A., Halman J.R., Wilson J., Aksimentiev A., Afonin K.A., Wanunu M. (2017). Picomolar Fingerprinting of Nucleic Acid Nanoparticles Using Solid-State Nanopores. ACS Nano.

[90] Raveendran M., Lee A.J., Wälti C., Actis P. (2018). Analysis of 2D DNA Origami with Nanopipettes. ChemElectroChem.

[91] Wang V., Ermann N., Keyser U.F. (2019). Current Enhancement in Solid-State Nanopores Depends on Three-Dimensional DNA Structure. Nano Lett..

[92] Zhu L., Zhang Z., Liu Q. (2020). Deformation-Mediated Translocation of DNA Origami Nanoplates through a Narrow Solid-State Nanopore. Anal. Chem..

[93] Yang J., Zhao N., Liang Y., Lu Z., Zhang C. (2021). Structure-Flexible DNA Origami Translocation through a Solid-State Nanopore. RSC Adv..

[94] He L., Charron M., Mensing P., Briggs K., Adams J., de Haan H., Tabard-Cossa V. (2023). DNA Origami Characterized via a Solid-State Nanopore: Insights into Nanostructure Dimensions, Rigidity and Yield. Nanoscale.

[95] Langer J., Jimenez de Aberasturi D., Aizpurua J., Alvarez-Puebla R.A., Auguié B., Baumberg J.J., Bazan G.C., Bell S.E.J., Boisen A., Brolo A.G. (2020). Present and Future of Surface-Enhanced Raman Scattering. ACS Nano.

[96] Radziuk D., Moehwald H. (2015). Prospects for Plasmonic Hot Spots in Single Molecule SERS towards the Chemical Imaging of Live Cells. Phys. Chem. Chem. Phys..

[97] Mühlschlegel P., Eisler H.-J., Martin O.J.F., Hecht B., Pohl D.W. (2005). Resonant Optical Antennas. Science.

[98] Huang C., Bouhelier A., Colas des Francs G., Bruyant A., Guenot A., Finot E., Weeber J.-C., Dereux A. (2008). Gain, Detuning, and Radiation Patterns of Nanoparticle Optical Antennas. Phys. Rev. B.

[99] Bharadwaj P., Deutsch B., Novotny L. (2009). Optical Antennas. Adv. Opt. Photonics.

[100] Wen B., Yang J., Hu C., Cai J., Zhou J. (2024). Top-Down Fabrication of Ordered Nanophotonic Structures for Biomedical Applications. Adv. Mater. Interfaces.

[101] Niu R., Shao J., Wu M., Liu C., Chao J. (2025). Single-Molecule Detection of Optical Signals Using DNA-Based Plasmonic Nanostructures. Biosensors.

[102] Qiu Y., Kuang C., Liu X., Tang L. (2022). Single-Molecule Surface-Enhanced Raman Spectroscopy. Sensors.

[103] Hanlon E.B., Manoharan R., Koo T.-W., Shafer K.E., Motz J.T., Fitzmaurice M., Kramer J.R., Itzkan I., Dasari R.R., Feld M.S. (2000). Prospects for in Vivo Raman Spectroscopy. Phys. Med. Biol..

[104] Guerrini L., Graham D. (2012). Molecularly-Mediated Assemblies of Plasmonic Nanoparticles for Surface-Enhanced Raman Spectroscopy Applications. Chem. Soc. Rev..

[105] Prinz J., Schreiber B., Olejko L., Oertel J., Rackwitz J., Keller A., Bald I. (2013). DNA Origami Substrates for Highly Sensitive Surface-Enhanced Raman Scattering. J. Phys. Chem. Lett..

[106] Kühler P., Roller E.-M., Schreiber R., Liedl T., Lohmüller T., Feldmann J. (2014). Plasmonic DNA-Origami Nanoantennas for Surface-Enhanced Raman Spectroscopy. Nano Lett..

[107] Thacker V.V., Herrmann L.O., Sigle D.O., Zhang T., Liedl T., Baumberg J.J., Keyser U.F. (2014). DNA Origami Based Assembly of Gold Nanoparticle Dimers for Surface-Enhanced Raman Scattering. Nat. Commun..

[108] Prinz J., Heck C., Ellerik L., Merk V., Bald I. (2016). DNA Origami Based Au–Ag-Core–Shell Nanoparticle Dimers with Single-Molecule SERS Sensitivity. Nanoscale.

[109] Simoncelli S., Roller E.-M., Urban P., Schreiber R., Turberfield A.J., Liedl T., Lohmüller T. (2016). Quantitative Single-Molecule Surface-Enhanced Raman Scattering by Optothermal Tuning of DNA Origami-Assembled Plasmonic Nanoantennas. ACS Nano.

[110] Tanwar S., Haldar K.K., Sen T. (2017). DNA Origami Directed Au Nanostar Dimers for Single-Molecule Surface-Enhanced Raman Scattering. J. Am. Chem. Soc..

[111] Zhan P., Wen T., Wang Z., He Y., Shi J., Wang T., Liu X., Lu G., Ding B. (2018). DNA Origami Directed Assembly of Gold Bowtie Nanoantennas for Single-Molecule Surface-Enhanced Raman Scattering. Angew. Chem. Int. Ed..

[112] Heck C., Kanehira Y., Kneipp J., Bald I. (2019). Amorphous Carbon Generation as a Photocatalytic Reaction on DNA-Assembled Gold and Silver Nanostructures. Molecules.

[113] Fang W., Jia S., Chao J., Wang L., Duan X., Liu H., Li Q., Zuo X., Wang L., Wang L. (2019). Quantizing Single-Molecule Surface-Enhanced Raman Scattering with DNA Origami Metamolecules. Sci. Adv..

[114] Niu R., Song C., Gao F., Fang W., Jiang X., Ren S., Zhu D., Su S., Chao J., Chen S. (2021). DNA Origami-Based Nanoprinting for the Assembly of Plasmonic Nanostructures with Single-Molecule Surface-Enhanced Raman Scattering. Angew. Chem. Int. Ed..

[115] Chikkaraddy R., Turek V.A., Lin Q., Griffiths J., de Nijs B., Keyser U.F., Baumberg J.J. (2021). Dynamics of Deterministically Positioned Single-bond Surface-enhanced Raman Scattering from DNA Origami Assembled in Plasmonic Nanogaps. J. Raman Spectrosc..

[116] Niu R., Gao F., Wang D., Zhu D., Su S., Chen S., YuWen L., Fan C., Wang L., Chao J. (2022). Pattern Recognition Directed Assembly of Plasmonic Gap Nanostructures for Single-Molecule SERS. ACS Nano.

[117] Kanehira Y., Tapio K., Wegner G., Kogikoski S., Rüstig S., Prietzel C., Busch K., Bald I. (2023). The Effect of Nanoparticle Composition on the Surface-Enhanced Raman Scattering Performance of Plasmonic DNA Origami Nanoantennas. ACS Nano.

[118] Kaur V., Tanwar S., Kaur G., Sen T. (2021). DNA-Origami-Based Assembly of Au@Ag Nanostar Dimer Nanoantennas for Label-Free Sensing of Pyocyanin. ChemPhysChem.

[119] Kaur V., Sharma M., Sen T. (2021). DNA Origami-Templated Bimetallic Nanostar Assemblies for Ultra-Sensitive Detection of Dopamine. Front. Chem..

[120] Fernanda Cardinal M., Rodríguez-González B., Alvarez-Puebla R.A., Pérez-Juste J., Liz-Marzán L.M. (2010). Modulation of Localized Surface Plasmons and SERS Response in Gold Dumbbells through Silver Coating. J. Phys. Chem. C.

[121] Kanehira Y., Kogikoski S., Titov E., Tapio K., Mostafa A., Bald I. (2024). Watching a Single Enzyme at Work Using Single-Molecule Surface-Enhanced Raman Scattering and DNA Origami-Based Plasmonic Antennas. ACS Nano.

[122] Dutta A., Tapio K., Suma A., Mostafa A., Kanehira Y., Carnevale V., Bussi G., Bald I. (2022). Molecular States and Spin Crossover of Hemin Studied by DNA Origami Enabled Single-Molecule Surface-Enhanced Raman Scattering. Nanoscale.

[123] Li G., Sun Z., Chen S., Lin J., Hao Q., Fan X., Qiu T. (2025). Surface-Enhanced Raman Scattering for Biosensing and Early Diagnosis. Appl. Phys. Lett..

[124] Shashkova S., Leake M.C. (2017). Single-Molecule Fluorescence Microscopy Review: Shedding New Light on Old Problems. Biosci. Rep..

[125] Radmacher N., Chizhik A.I., Nevskyi O., Gallea J.I., Gregor I., Enderlein J. (2025). Molecular Level Super-Resolution Fluorescence Imaging. Annu. Rev. Biophys..

[126] Jungmann R., Steinhauer C., Scheible M., Kuzyk A., Tinnefeld P., Simmel F.C. (2010). Single-Molecule Kinetics and Super-Resolution Microscopy by Fluorescence Imaging of Transient Binding on DNA Origami. Nano Lett..

[127] Johnson-Buck A., Nangreave J., Kim D.-N., Bathe M., Yan H., Walter N.G. (2013). Super-Resolution Fingerprinting Detects Chemical Reactions and Idiosyncrasies of Single DNA Pegboards. Nano Lett..

[128] Schnitzbauer J., Strauss M.T., Schlichthaerle T., Schueder F., Jungmann R. (2017). Super-Resolution Microscopy with DNA-PAINT. Nat. Protoc..

[129] Wade O.K., Woehrstein J.B., Nickels P.C., Strauss S., Stehr F., Stein J., Schueder F., Strauss M.T., Ganji M., Schnitzbauer J. (2019). 124-Color Super-Resolution Imaging by Engineering DNA-PAINT Blinking Kinetics. Nano Lett..

[130] Jungmann R., Avendaño M.S., Woehrstein J.B., Dai M., Shih W.M., Yin P. (2014). Multiplexed 3D Cellular Super-Resolution Imaging with DNA-PAINT and Exchange-PAINT. Nat. Methods.

[131] Jungmann R., Avendaño M.S., Dai M., Woehrstein J.B., Agasti S.S., Feiger Z., Rodal A., Yin P. (2016). Quantitative Super-Resolution Imaging with QPAINT. Nat. Methods.

[132] Reinhardt S.C.M., Masullo L.A., Baudrexel I., Steen P.R., Kowalewski R., Eklund A.S., Strauss S., Unterauer E.M., Schlichthaerle T., Strauss M.T. (2023). Ångström-Resolution Fluorescence Microscopy. Nature.

[133] Xu W., Yin P., Dai M. (2018). Super-resolution Geometric Barcoding for Multiplexed MiRNA Profiling. Angew. Chem. Int. Ed..

[134] Kocabey S., Chiarelli G., Acuna G.P., Ruegg C. (2023). Ultrasensitive and Multiplexed MiRNA Detection System with DNA-PAINT. Biosens. Bioelectron..

[135] Loretan M., Barella M., Fuchs N., Kocabey S., Kołątaj K., Stefani F.D., Acuna G.P. (2025). Direct Single-Molecule Detection and Super-Resolution Imaging with a Low-Cost Portable Smartphone-Based Microscope. Nat. Commun..

[136] Selnihhin D., Sparvath S.M., Preus S., Birkedal V., Andersen E.S. (2018). Multifluorophore DNA Origami Beacon as a Biosensing Platform. ACS Nano.

[137] Domljanovic I., Loretan M., Kempter S., Acuna G.P., Kocabey S., Ruegg C. (2022). DNA Origami Book Biosensor for Multiplex Detection of Cancer-Associated Nucleic Acids. Nanoscale.

[138] Grabenhorst L., Pfeiffer M., Schinkel T., Kümmerlin M., Brüggenthies G.A., Maglic J.B., Selbach F., Murr A.T., Tinnefeld P., Glembockyte V. (2025). Engineering Modular and Tunable Single-Molecule Sensors by Decoupling Sensing from Signal Output. Nat. Nanotechnol..

[139] Tsang E., Lund L.M., Birkedal V., Gothelf K.V. (2026). Single-Molecule Nucleic Acid Detection with a Reconfigurable Rotating DNA Origami Nanodevice. ACS Nano.

[140] Acuna G.P., Möller F.M., Holzmeister P., Beater S., Lalkens B., Tinnefeld P. (2012). Fluorescence Enhancement at Docking Sites of DNA-Directed Self-Assembled Nanoantennas. Science.

[141] Ochmann S.E., Vietz C., Trofymchuk K., Acuna G.P., Lalkens B., Tinnefeld P. (2017). Optical Nanoantenna for Single Molecule-Based Detection of Zika Virus Nucleic Acids without Molecular Multiplication. Anal. Chem..

[142] Trofymchuk K., Glembockyte V., Grabenhorst L., Steiner F., Vietz C., Close C., Pfeiffer M., Richter L., Schütte M.L., Selbach F. (2021). Addressable Nanoantennas with Cleared Hotspots for Single-Molecule Detection on a Portable Smartphone Microscope. Nat. Commun..

[143] Pfeiffer M., Trofymchuk K., Ranallo S., Ricci F., Steiner F., Cole F., Glembockyte V., Tinnefeld P. (2021). Single Antibody Detection in a DNA Origami Nanoantenna. iScience.

[144] Yaadav R., Trofymchuk K., Dass M., Behrendt V., Hauer B., Schütz J., Close C., Scheckenbach M., Ferrari G., Mäurer L. (2025). Bringing Attomolar Detection to the Point-of-Care with Nanopatterned DNA Origami Nanoantennas. Adv. Mater..

[145] Roy R., Hohng S., Ha T. (2008). A Practical Guide to Single-Molecule FRET. Nat. Methods.

[146] Andersen E.S., Dong M., Nielsen M.M., Jahn K., Subramani R., Mamdouh W., Golas M.M., Sander B., Stark H., Oliveira C.L.P. (2009). Self-Assembly of a Nanoscale DNA Box with a Controllable Lid. Nature.

[147] Zadegan R.M., Jepsen M.D.E., Thomsen K.E., Okholm A.H., Schaffert D.H., Andersen E.S., Birkedal V., Kjems J. (2012). Construction of a 4 Zeptoliters Switchable 3D DNA Box Origami. ACS Nano.

[148] Zadegan R.M., Jepsen M.D.E., Hildebrandt L.L., Birkedal V., Kjems J. (2015). Construction of a Fuzzy and Boolean Logic Gates Based on DNA. Small.

[149] Tang M.S.L., Shiu S.C.-C., Godonoga M., Cheung Y.-W., Liang S., Dirkzwager R.M., Kinghorn A.B., Fraser L.A., Heddle J.G., Tanner J.A. (2018). An Aptamer-Enabled DNA Nanobox for Protein Sensing. Nanomed. Nanotechnol. Biol. Med..

[150] Marras A.E., Zhou L., Su H.-J., Castro C.E. (2015). Programmable Motion of DNA Origami Mechanisms. Proc. Natl. Acad. Sci. USA.

[151] Anger P., Bharadwaj P., Novotny L. (2006). Enhancement and Quenching of Single-Molecule Fluorescence. Phys. Rev. Lett..

[152] Vietz C., Lalkens B., Acuna G.P., Tinnefeld P. (2016). Functionalizing Large Nanoparticles for Small Gaps in Dimer Nanoantennas. New J. Phys..

[153] Puchkova A., Vietz C., Pibiri E., Wünsch B., Sanz Paz M., Acuna G.P., Tinnefeld P. (2015). DNA Origami Nanoantennas with over 5000-Fold Fluorescence Enhancement and Single-Molecule Detection at 25 ΜM. Nano Lett..

[154] Glembockyte V., Grabenhorst L., Trofymchuk K., Tinnefeld P. (2021). DNA Origami Nanoantennas for Fluorescence Enhancement. Acc. Chem. Res..

[155] Vietz C., Kaminska I., Sanz Paz M., Tinnefeld P., Acuna G.P. (2017). Broadband Fluorescence Enhancement with Self-Assembled Silver Nanoparticle Optical Antennas. ACS Nano.

[156] Close C., Trofymchuk K., Grabenhorst L., Lalkens B., Glembockyte V., Tinnefeld P. (2022). Maximizing the Accessibility in DNA Origami Nanoantenna Plasmonic Hotspots. Adv. Mater. Interfaces.

[157] Grabenhorst L., Trofymchuk K., Steiner F., Glembockyte V., Tinnefeld P. (2020). Fluorophore Photostability and Saturation in the Hotspot of DNA Origami Nanoantennas. Methods Appl. Fluoresc..

[158] Linko V., Keller A. (2023). Stability of DNA Origami Nanostructures in Physiological Media: The Role of Molecular Interactions. Small.

[159] Montoya R., Deckerman P., Guler M.O. (2025). Protein Recognition Methods for Diagnostics and Therapy. BBA Adv..

[160] Shin S., Kim J., Song E., Han S., Hohng S. (2025). Analytical Techniques for Nucleic Acid and Protein Detection with Single-Molecule Sensitivity. Exp. Mol. Med..

[161] Brown A., Brill J., Amini R., Nurmi C., Li Y. (2024). Development of Better Aptamers: Structured Library Approaches, Selection Methods, and Chemical Modifications. Angew. Chem. Int. Ed..

[162] Deng B., Lin Y., Wang C., Li F., Wang Z., Zhang H., Li X.-F., Le X.C. (2014). Aptamer Binding Assays for Proteins: The Thrombin Example—A Review. Anal. Chim. Acta.

[163] Shetty R.M., Brady S.R., Rothemund P.W.K., Hariadi R.F., Gopinath A. (2021). Bench-Top Fabrication of Single-Molecule Nanoarrays by DNA Origami Placement. ACS Nano.

[164] Ijäs H., Trommler J., Nguyen L., van Rest S., Nickels P.C., Liedl T., Urban M.J. (2025). DNA Origami Signal Amplification in Lateral Flow Immunoassays. Nat. Commun..

[165] Nickels P.C., Høiberg H.C., Simmel S.S., Holzmeister P., Tinnefeld P., Liedl T. (2016). DNA Origami Seesaws as Comparative Binding Assay. ChemBioChem.

[166] Ghamari S., Chiarelli G., Kołątaj K., Subramanian S., Acuna G.P., Vollmer F. (2025). Label-Free (Fluorescence-Free) Sensing of a Single DNA Molecule on DNA Origami Using a Plasmon-Enhanced WGM Sensor. Nanophotonics.

[167] Koirala D., Shrestha P., Emura T., Hidaka K., Mandal S., Endo M., Sugiyama H., Mao H. (2014). Single-Molecule Mechanochemical Sensing Using DNA Origami Nanostructures. Angew. Chem. Int. Ed..

[168] Büber E., Yaadav R., Schröder T., Franquelim H.G., Tinnefeld P. (2024). DNA Origami Vesicle Sensors with Triggered Single-Molecule Cargo Transfer. Angew. Chem. Int. Ed..

[169] Daems D., Rutten I., Bath J., Decrop D., Van Gorp H., Ruiz E.P., De Feyter S., Turberfield A.J., Lammertyn J. (2019). Controlling the Bioreceptor Spatial Distribution at the Nanoscale for Single Molecule Counting in Microwell Arrays. ACS Sens..

[170] Jeon B., Guareschi M.M., Stewart J.M., Wu E., Gopinath A., Arroyo-Currás N., Dauphin-Ducharme P., Plaxco K.W., Lukeman P.S., Rothemund P.W.K. (2025). Modular DNA Origami–Based Electrochemical Detection of DNA and Proteins. Proc. Natl. Acad. Sci. USA.

[171] Kuzyk A., Schreiber R., Zhang H., Govorov A.O., Liedl T., Liu N. (2014). Reconfigurable 3D Plasmonic Metamolecules. Nat. Mater..

[172] Funck T., Nicoli F., Kuzyk A., Liedl T. (2018). Sensing Picomolar Concentrations of RNA Using Switchable Plasmonic Chirality. Angew. Chem. Int. Ed..

[173] Li C., He T., Yang X., Feng C., Zhang Z., Zhu J., Dong S., Shi Y., Wei Z., Jiao H. (2024). Enhanced Circular Dichroism for Achiral Sensing Based on a DNA-Origami-Empowered Anapole Metasurface. Nano Lett..

[174] Huang Y., Nguyen M.-K., Natarajan A.K., Nguyen V.H., Kuzyk A. (2018). A DNA Origami-Based Chiral Plasmonic Sensing Device. ACS Appl. Mater. Interfaces.

[175] Sannohe Y., Endo M., Katsuda Y., Hidaka K., Sugiyama H. (2010). Visualization of Dynamic Conformational Switching of the G-Quadruplex in a DNA Nanostructure. J. Am. Chem. Soc..

[176] Subramani R., Juul S., Rotaru A., Andersen F.F., Gothelf K.V., Mamdouh W., Besenbacher F., Dong M., Knudsen B.R. (2010). A Novel Secondary DNA Binding Site in Human Topoisomerase I Unravelled by Using a 2D DNA Origami Platform. ACS Nano.

[177] Chao J., Zhang P., Wang Q., Wu N., Zhang F., Hu J., Fan C.H., Li B. (2016). Single-Molecule Imaging of DNA Polymerase I (Klenow Fragment) Activity by Atomic Force Microscopy. Nanoscale.

[178] Zhang P., Liu X., Liu P., Wang F., Ariyama H., Ando T., Lin J., Wang L., Hu J., Li B. (2020). Capturing Transient Antibody Conformations with DNA Origami Epitopes. Nat. Commun..

[179] Tintoré M., Gállego I., Manning B., Eritja R., Fàbrega C. (2013). DNA Origami as a DNA Repair Nanosensor at the Single-Molecule Level. Angew. Chem. Int. Ed..

[180] Hernández-Ainsa S., Misiunas K., Thacker V.V., Hemmig E.A., Keyser U.F. (2014). Voltage-Dependent Properties of DNA Origami Nanopores. Nano Lett..

[181] Li C.-Y., Hemmig E.A., Kong J., Yoo J., Hernández-Ainsa S., Keyser U.F., Aksimentiev A. (2015). Ionic Conductivity, Structural Deformation, and Programmable Anisotropy of DNA Origami in Electric Field. ACS Nano.

[182] Ketterer P., Ananth A.N., Laman Trip D.S., Mishra A., Bertosin E., Ganji M., van der Torre J., Onck P., Dietz H., Dekker C. (2018). DNA Origami Scaffold for Studying Intrinsically Disordered Proteins of the Nuclear Pore Complex. Nat. Commun..

[183] Wen C., Bertosin E., Shi X., Dekker C., Schmid S. (2023). Orientation-Locked DNA Origami for Stable Trapping of Small Proteins in the Nanopore Electro-Osmotic Trap. Nano Lett..

[184] Pal S., Naik A., Rao A., Chakraborty B., Varma M.M. (2022). Aptamer-DNA Origami-Functionalized Solid-State Nanopores for Single-Molecule Sensing of G-Quadruplex Formation. ACS Appl. Nano Mater..

[185] Funke J.J., Ketterer P., Lieleg C., Schunter S., Korber P., Dietz H. (2016). Uncovering the Forces between Nucleosomes Using DNA Origami. Sci. Adv..

[186] Le J.V., Luo Y., Darcy M.A., Lucas C.R., Goodwin M.F., Poirier M.G., Castro C.E. (2016). Probing Nucleosome Stability with a DNA Origami Nanocaliper. ACS Nano.

[187] Funke J.J., Dietz H. (2016). Placing Molecules with Bohr Radius Resolution Using DNA Origami. Nat. Nanotechnol..

[188] Marras A.E., Shi Z., Lindell M.G., Patton R.A., Huang C.-M., Zhou L., Su H.-J., Arya G., Castro C.E. (2018). Cation-Activated Avidity for Rapid Reconfiguration of DNA Nanodevices. ACS Nano.

[189] Shi Z., Arya G. (2020). Free Energy Landscape of Salt-Actuated Reconfigurable DNA Nanodevices. Nucleic Acids Res..

[190] Darcy M., Crocker K., Wang Y., Le J.V., Mohammadiroozbahani G., Abdelhamid M.A.S., Craggs T.D., Castro C.E., Bundschuh R., Poirier M.G. (2022). High-Force Application by a Nanoscale DNA Force Spectrometer. ACS Nano.

[191] Centola M., Poppleton E., Ray S., Centola M., Welty R., Valero J., Walter N.G., Šulc P., Famulok M. (2024). A Rhythmically Pulsing Leaf-Spring DNA-Origami Nanoengine That Drives a Passive Follower. Nat. Nanotechnol..

[192] Pensa E., Bogawat Y., Simmel F.C., Santiago I. (2022). Single DNA Origami Detection by Nanoimpact Electrochemistry. ChemElectroChem.

[193] Nickels P.C., Wünsch B., Holzmeister P., Bae W., Kneer L.M., Grohmann D., Tinnefeld P., Liedl T. (2016). Molecular Force Spectroscopy with a DNA Origami–Based Nanoscopic Force Clamp. Science.

[194] Brockman J.M., Su H., Blanchard A.T., Duan Y., Meyer T., Quach M.E., Glazier R., Bazrafshan A., Bender R.L., Kellner A.V. (2020). Live-Cell Super-Resolved PAINT Imaging of Piconewton Cellular Traction Forces. Nat. Methods.

[195] Hu Y., Rogers J., Duan Y., Velusamy A., Narum S., Al Abdullatif S., Salaita K. (2024). Quantifying T Cell Receptor Mechanics at Membrane Junctions Using DNA Origami Tension Sensors. Nat. Nanotechnol..

[196] Ohshiro T., Taniguchi M. (2022). Review of the Use of Nanodevices to Detect Single Molecules. Anal. Biochem..

[197] Barman S.M., Parakh A., Leema A.A., Balakrishnan P., Avthankar A., Tulaskar D.P., Assudani P.J., Nemane S., Rewatkar P., Kulkarni M.B. (2025). Single-Molecule Detection Technologies: Advances in Devices, Transduction Mechanisms, and Functional Materials for Real-World Biomedical and Environmental Applications. Biosensors.

